# Computer models predict differential dendritic vulnerability with ischemia and spreading depression

**DOI:** 10.1371/journal.pcbi.1014701

**Published:** 2026-09-08

**Authors:** Adam J. H. Newton, William W. Lytton, Marcello DiStasio, Robert A. McDougal

**Affiliations:** 1 Department of Biostatistics, Yale School of Public Health, New Haven, Connecticut, United States of America; 2 Department of Physiology and Pharmacology, SUNY Downstate Health Sciences University, Brooklyn, New York, United States of America; 3 Department of Neurology, Kings County Hospital Center, Brooklyn, New York, United States of America; 4 Department of Pathology, Yale School of Medicine, New Haven, Connecticut, United States of America; 5 Department of Ophthalmology and Visual Science, Yale School of Medicine, New Haven, Connecticut, United States of America; 6 Wu Tsai Institute, Yale University, New Haven, Connecticut, United States of America; 7 Department of Biomedical Informatics and Data Science, Yale School of Medicine, New Haven, Connecticut, United States of America; 8 Program in Computational Biology and Bioinformatics, Yale University, New Haven, Connecticut, United States of America; 9 Interdepartmental Neuroscience Program, Yale University, New Haven, Connecticut, United States of America; Swiss Federal Institute of Technology Zurich Institute of Neuroinformatics: Institut fur Neuroinformatik UZH/ETH, SWITZERLAND

## Abstract

Ischemia, whether abrupt or chronic, limits ATP production and disrupts ATP-dependent homeostatic mechanisms, leading to alterations in both intracellular and extracellular ion concentrations. Inadequate neuronal ATP triggers K^+^ release and increased extracellular K^+^ depolarizes neurons, leading to additional K^+^ release; this positive feedback phenomenon is known as spreading depolarization (SD). When the depolarizing effects are strong enough, the cells undergo depolarization blockade, known as spreading depression. Excess extracellular K^+^ increases energy demand from the Na^+^-K^+^ pump, producing a pathological confluence of increased demand with reduced delivery of energy. The resulting changes have profound effects at subcellular, cellular, and network scales of brain function. We hypothesized that consequences of ischemic or SD homeostatic failure would differ on the subcellular scale, with differences between disjunct dendritic regions of a hippocampal CA1 pyramidal neuron. To evaluate the interplay between morphology and ion concentrations, we used a mechanistic simulation incorporating neuronal morphology, pumps, exchangers, voltage-, and Ca^2+^-sensitive ion channels. In both cases, calcium accumulation was greatest in the basal dendrites, suggesting these dendrites would show the greatest effects of excitotoxicity. By contrast, in ischemia, but not in SD, distal apical dendrites were exposed to greater intracellular chloride concentrations, which may lead to dendritic beading.

## Author summary

Neurons are cells, and like all cells, they respond to changes in their environment. Different conditions, such as ischemia and elevated potassium concentrations, can trigger a response known as spreading depression, where the membrane potential of the neuron is depolarized and the cell is unable to generate action potentials. To better understand the cellular response to these two conditions, we constructed a detailed simulation study incorporating a neuron, an astrocyte, and the nearby extracellular space. The astrocyte used a simplified morphology, while the neuron was considered in full morphological detail to examine how its different regions responded. Both cell models included ion channels, homeostatic mechanisms, and dynamic chemical concentrations. In both conditions, our model found that calcium concentration was most elevated in the basal dendrites. Ischemia led to elevated intracellular chloride concentrations in distal apical dendrites, while elevated potassium concentrations did not. Elevated calcium is associated with excitotoxicity, and elevated chloride has been associated with dendritic beading.

## 1. Introduction

Continuous oxygen delivery provides the ATP needed to maintain ion homeostasis and maintain tissue viability, providing energy for intracellular and neuron-to-neuron signaling that drive brain function [1]. Most of the energy consumption of neural tissue goes to the 3:2 Na^+^-K^+^ pump to drive these ions against their gradients [2]. The time course of an energy deficit at the pump will determine the risk of cell death and the nature of cell damage [3,4]. Many other neuronal homeostatic mechanisms either directly consume ATP as well or are exchangers leveraging the gradients established by the ATP-dependent Na^+^-K^+^ pump.

Ionic homeostasis maintains a variety of gradients, sustaining various aspects of cell function, which may fail at different times and to different extents following ATP-depletion, producing varying spatiotemporal failure patterns with distinct patterns of ion failure, with different subcellular and tissue pathology patterns. For example, Na^+^ and K^+^ gradients are required for action potential generation and transmission; the Ca^2+^ gradient is required for synaptic transmission [5]. The Ca^2+^ gradient is maintained by two ATP pumps, plasma membrane Ca^2+^ ATPase (PMCA), and the sarco-endoplasmic reticulum Ca^2+^ ATPase (SERCA), overall with far less energy demand than the Na^+^-K^+^ pump [6]. ${\text{Ca}}_{cyt}^{2+}$ is also extruded from the neuron by Na^+^-Ca^2+^ exchange, utilizing the Na^+^ gradient. Cl^-^ symporters exploit the gradient created by the Na^+^-K^+^ pump, without directly consuming any ATP, to move Cl^-^ in or out of the cell [7].

Spreading depolarization (SD), seen in a wide range of neurological diseases, is a wave of neuronal depolarization associated with changes in extracellular ionic concentrations, particularly in K^+^ and Na^+^. SD propagates through neural tissue at ~1−8 mm/min [8–10]. Because ionic concentration homeostasis requires energy, ionic shifts and energy deficit produces a pathological synergy: SD augments local ischemia, and ischemia prevents recovery from SD. Transient ischemia will trigger SD and worsen ischemia. Spatiotemporal interactions between SD and oxygen delivery creates the heterogeneous tissue damage seen in patients with SD-associated disorders, depending on the duration and metabolic capacity of the tissue. SD is also associated with traumatic brain injury, tumor, epilepsy, and ischemic stroke and can be associated with MRI anomalies in all of these disorders, sometimes far from the primary site of injury [3].

The frequency and duration of SDs following an ischemic or hemorrhagic stroke, traumatic injury, or growth of a tumor or other expanding lesion can serve as a biomarker for the metabolic state of the tissue and suggest secondary therapy [11]. The ubiquity of SD in multiple pathologies indicates its relevance as a target for clinical intervention aimed at avoiding a threatened pattern and pace of damage expected from predicted pathophysiology [12].

At durations and severities of ischemia insufficient to cause acute cell death through phospholipase activation, lipid peroxidation, cytoskeletal collapse, and plasma membrane rupture due to osmotic swelling, ischemia leads to non-lethal cell damage. This damage is manifested histologically as dendritic beading, where water enters – partially through Cl^-^ symporters (NKCC1 and KCC2) [13,14] – causing varicosities and spine loss, or outright dendritic loss if there is also elevated ${\text{Ca}}_{cyt}^{2+}$ from excess glutamate [4,15]. Dendritic loss can occur late following beading [16,17] or may occur more immediately due to Ca^2+^ entry causing excitotoxicity. The microanatomical patterns of dendritic damage, loss, and of cell death depend on the details of the underlying causes.

We hypothesized that SD and ischemia, in various combinations of severity, will lead to predictable spatiotemporal patterns of damage which can predict the extent of prolonged and extended homeostatic failure and thus guide therapy. To test this, we used a model pyramidal cortical neuron with detailed morphology, intracellular Ca^2+^ dynamics [18,19], glutamate activation [20] and ion pumps [21,22]. We also included a single simplified astrocyte cell model, represented as a line of compartments parallel to the neuron model. The astrocyte model also consumed energy to clear glutamate [20] and K^+^ [23–25].

The model parameters were tuned to produce physiological spike rates and maintain homeostasis under simulated current clamp. Subsequently, we simulated three conditions: (1) elevated extracellular K^+^ concentration ($[K^{+}{]}_{o}$), causing SD alone as would be seen in migraine aura; (2) hypoxia/ischemia at baseline $[K^{+}{]}_{o}$, as in ischemic tissue that may generate secondary SD; and (3) ischemia with elevated $[K^{+}{]}_{o}$, as in hypoxic spreading depolarization (HSD).

We contrasted the effects of SD and hypoxia: hypoxia, but not SD alone, caused a delayed depolarization resulting from the replacement of intracellular K^+^ with Na^+^, and also produced secondary changes to Ca^2+^ and Cl^-^. Additionally, distinct subcellular zones of vulnerability were found, with highest susceptibility to ischemia-induced excitotoxicity in basal dendrites, contrasted with greater susceptibility of apical dendrites to increased cytoplasmic Cl^-^ that may underlie acute dendritic beading. These distinctive damage patterns would provide a pathological signature reflecting different types of cell damage and varying risks of cell-death.

## 2. Results

### 2.1. Dendrite beading is observed in the core and penumbra of human cortical infarct

**Fig 1:**
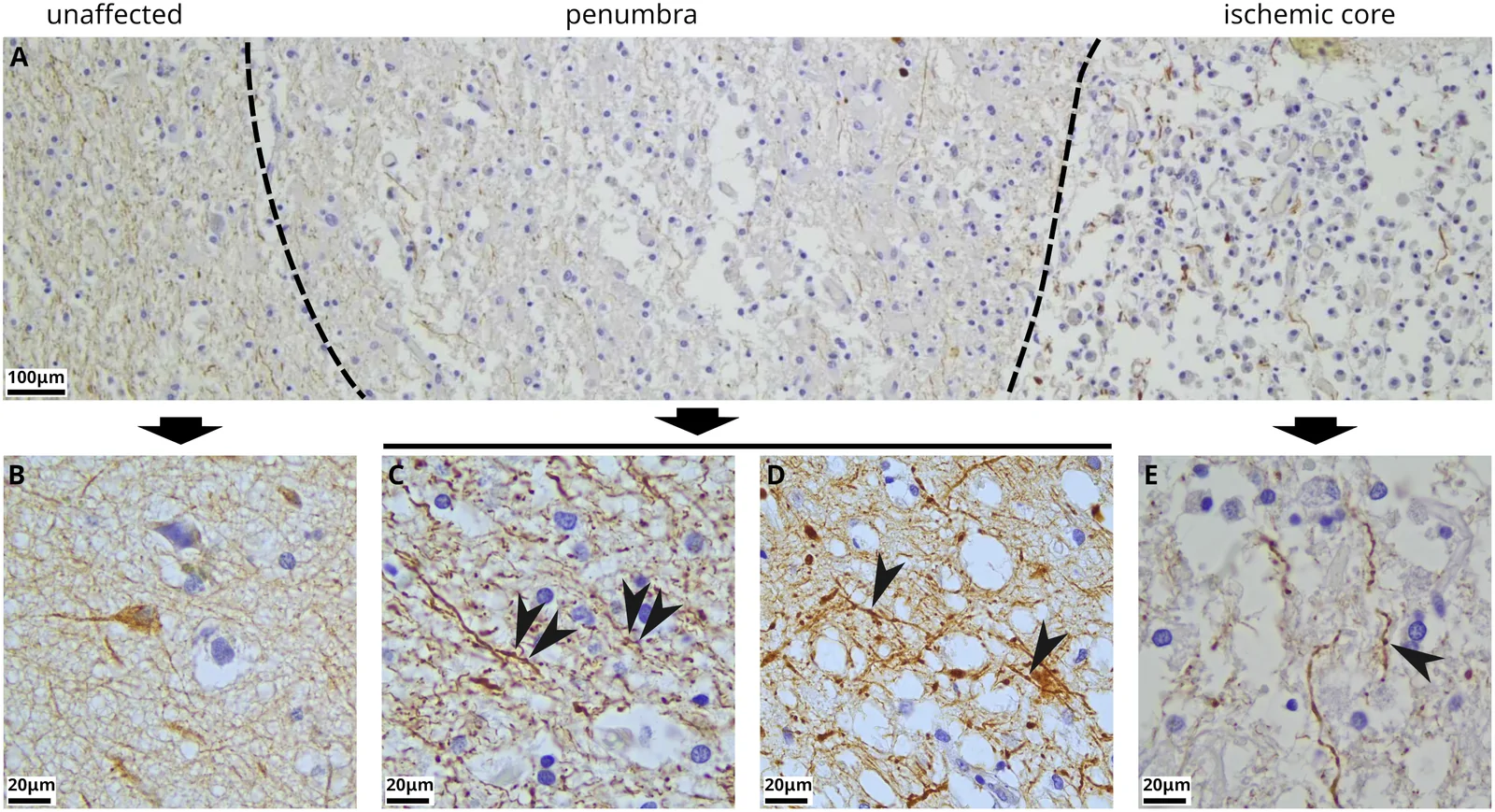
An example of neurite varicosities (“beading”) in the penumbra and the core of human cortical infarct, as shown with immunohistochemistry for neurofilament. **(A)** Low-power view demonstrating the zones of brain tissue affected by subacute infarct. At left, in a region of the gray-white matter junction (with occasional layer VI neurons) that is relatively spared of ischemic injury, there is more organized neuropil occupied by neurons, oligodendroglia, and astrocytes. In the penumbra of the infarct, there is vacuolization of the neuropil and infiltration by macrophages, with some loss of tissue integrity. In the ischemic core, there is severe loss of brain tissue, with cavitation and near-replacement of neuropil by macrophages and only disorganized and dissociated neurites surviving, with prominent swelling. **(B)** Enlarged view of unaffected tissue with no visible neurite varicosities. **(C,D)** Enlarged view of penumbra with arrowheads indicating examples of neurite varicosities. **(E)** Enlarged view of the ischemic core with arrowhead indicating an example of neurite varicosity.

Neuropathologic review of post-mortem human brain tissue from three subjects of ages 62–75 years with subacute infarcts of cerebral cortex was performed using immunohistochemistry for neurofilament (abundant heteropolymer neural proteins that contribute to maintenance of neuronal structure). As expected, beading, characterized by periodic varicosities (i.e., swollen foci containing neurofilament) along the length of neurites was observed in both the penumbra and within the ischemic core of the infarcted tissue, but not in adjacent uninvolved cortex (Fig 1).

Histologic sections are static and thin (5 μm) slices and thus only show a snapshot of small parts of the dendritic arbor. We sought a more comprehensive dynamic understanding of 1) the mechanisms of SD and hypoxic SD (‘HSD’) in humans, 2) the extent to which they drive ischemic damage, and 3) how neurite morphology contributes to vulnerability. We therefore developed multiscale 3-dimensional models of pyramidal neurons with simulated SD and HSD.

### 2.2. Membrane potential changes under hypoxia were slower but larger than those due to elevated extracellular potassium

We hypothesized that the high density of dendrites near the soma would give rise to different vulnerabilities in different parts of the dendritic arbor under these conditions. In particular, we hypothesized that the densely packed basal dendrites would be more vulnerable due to positive feedback loops from other dendrites where increased extracellular K^+^ or glutamate would cause neighboring dendrites to depolarize, releasing more K^+^, while also increasing the demand on the Na^+^-K^+^ pump consuming more ATP reducing the ability for the dendrite to respond to further stress.

**Fig 2:**
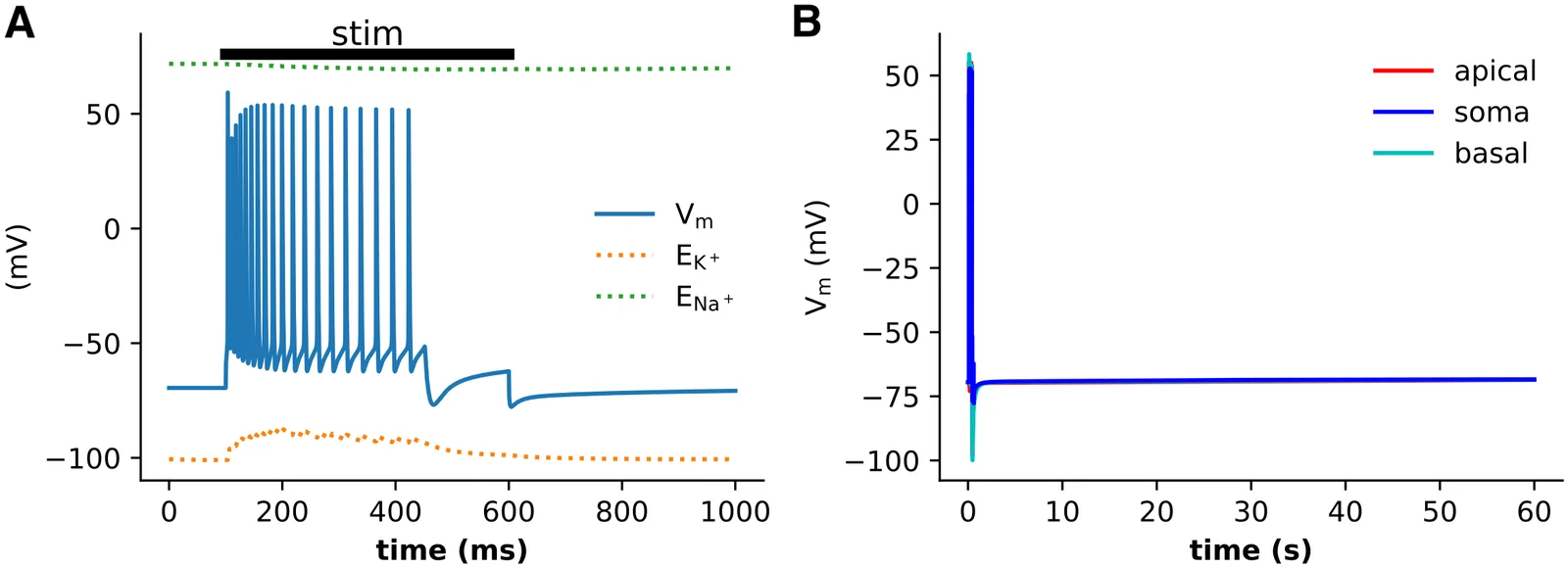
Control: membrane potential returns to baseline following 500 ms somatic current stimulation. **(A)** The first 0.5 s of somatic membrane potentials shows a burst of APs followed by depolarization block and quick return to RMP at the end of the stimulus. Nernst potentials remain relatively static, with a small rise in ${\text{E}}_{K}$ from $[K^{+}{]}_{o}$ changes due to APs. **(B)** Following the burst of APs that depolarized all neuronal compartments, membrane homeostasis was rapidly restored and maintained indefinitely – 1 min of simulation shown to demonstrate the stability of the RMP.

Under physiological conditions, neurons respond to moderate depolarizing stressors with a physiologically rapid return to baseline resting membrane potential (RMP) at the end of the burst (Fig 2). Following a common experimental protocol [26,27], we depolarized the neuron with a 500 ms long 1.0 nA current injection. This current produced a large burst of APs resulting in large ionic fluxes, with the influx of Na^+^ and efflux of K^+^. Homeostasis was quickly restored in all compartments, as pumps quickly restored ion concentrations and recovered Nernst potentials. By their nature and by the distribution of ions, even when the cell is at rest, sodium channels admit a small amount of sodium into the cell, and potassium channels release a small of potassium to the extracellular space. Under normoxic conditions, pumps compensate for these ion fluxes, and the concentrations and corresponding Nernst potentials are held approximately constant (dashed lines in Fig 2A). Following current injection, there was little change in membrane potential (${\text{V}}_{m}$, the difference between the electric charge inside and outside the compartment) throughout the neuron for the rest of the simulation (Fig 2B).

**Fig 3:**
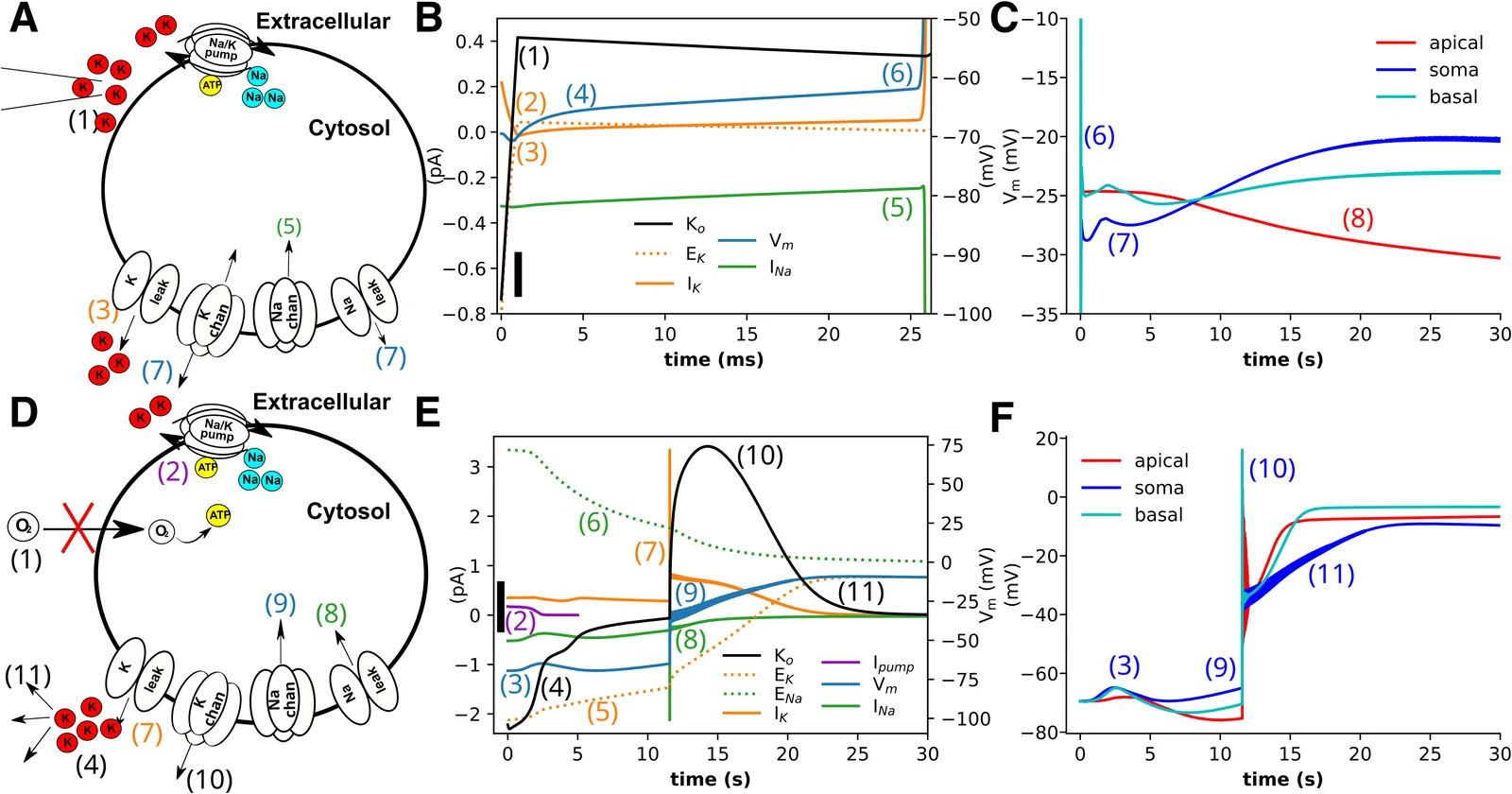
Either elevated extracellular K^+^ or hypoxia gave rise to sustained depolarizations, $↑[{𝐊}^{+}{]}_{𝐨}$ produced rapid depolarizations, whereas hypoxia led to slower but larger depolarizations. **(A-C)** Elevated extracellular K^+^ causes a spike followed by sustained depolarization. Sequence of events (numbers on panels): (1) elevation of extracellular K^+^ from 2.9 mM to 10 mM; (2) ${\text{E}}_{K}$ increase above the ${\text{V}}_{m}$; (3) $I_{K}$ leak reverses; (4) neuron depolarizes; (5) ${\text{V}}_{m}$ approaches spike threshold (~−60 mV); (6) producing the upswing of AP; (7) at the end of the spike, continued Na^+^ currents create a new equilibrium. (8) apical ${\text{V}}_{m}$ falls while somatic and basal rise, with small high frequency oscillation. **(A)** Schematic. **(B)** Currents $I_{K}$ and $I_{Na}$ are shown the left axis, potentials ${\text{E}}_{K}$ and ${\text{V}}_{m}$ on the right axis. Changes occurred rapidly, on a millisecond timescale. Scale bar of 1 mM applies to $[K^{+}{]}_{o}$ dynamics. **(C)** A single AP (truncated y-axis) is followed by incomplete repolarization and sustained depolarization consistent with SD. Regional variations in ${\text{V}}_{m}$ result from extracellular differences in O_2_ and K^+^ arising from the amplifying effects of interacting dendrites near the soma. Concentrations in a compartment of the basal dendrites 201 μm, and apical dendrites 611 μm from the soma are shown for comparison. **(D-F)** Another route to SD: hypoxia reduces Na^+^-K^+^ pump activity and ion homeostasis fails. Hypoxia led to a slower breakdown in homeostasis than elevated K^+^, hence the longer timescales shown in **(E&F)**. Sequence of events: (1) ↓ O_2_ →↓ ATP; (2) ↓ Na^+^-K^+^ pump; (3) $↑{\text{V}}_{m}$; (4) $↑[K^{+}{]}_{o}$; (5) $↑{\text{E}}_{K}$; (6) $↓{\text{E}}_{Na}$; (7) $↓I_{K}$; (8) $↓I_{Na}$; (9) ${\text{V}}_{m}$ approaches spike threshold producing a partial AP; (10) large $I_{K}$ led to greater accumulation extracellular K^+^ and further sustained depolarization with small high-frequency oscillations; (11) excess extracellular K^+^ diffuses away as the neuron and ${\text{V}}_{m}$ remains depolarized as K^+^ is replaced with Na^+^. **(D)** Schematic. **(E)** Currents $I_{K}$ and $I_{Na}$ are shown on the left axis, excluding the contribution of Na^+^-K^+^ pump; potentials ${\text{E}}_{K}$, ${\text{E}}_{Na}$, and ${\text{V}}_{m}$ on the right axis; $[K^{+}{]}_{o}$ 2.9 to 5.9; scale bar of 0.5 mM for $[K^{+}{]}_{o}$ concentrations is half the change of the scale bar in **B. (F)** The variation in ${\text{V}}_{m}$ between the soma and the dendrites. A compartment of the basal dendrites 201 μm, and apical dendrites 611 μm from the soma, are shown for comparison.

Elevated extracellular K^+^ rapidly produced a sustained depolarization with variation on ${\text{V}}_{m}$ in the entire cell, since depolarization occurred throughout the dendritic tree, eliminating the dendrites as current sinks (Fig 3A–3C). (1) We ramped up extracellular K^+^ to 10 mM in the first 1 ms; (Fig 3B) (2) ${\text{E}}_{K}$ followed K^+^  passing ${\text{V}}_{m}$, (3) thereby reducing and even briefly reversing $I_{K}$ to an inward current (4). This depolarized the cell towards the spike threshold (5) producing the sudden downward (inward) $I_{Na}$ deflection (where Na^+^ $m_{\infty }$ hits inflection) (6) triggering a single AP. (7) Incomplete repolarization to a sustained depolarization was consistent with SD (Fig 3C). (8) During the sustained depolarization, there were relatively small changes in ${\text{V}}_{m}$, which were different for different parts of the neuron. Notably, apical ${\text{V}}_{m}$ declined, as there was less competition for available O_2_ in the apical than in the dense dendritic arbor near the soma.

By contrast, sustained hypoxia resulted in delayed depolarization (Fig 3E–3F). A relatively small initial change in ${\text{V}}_{m}$ (3) masked substantial shifts in Nernst potentials: ${\text{E}}_{Na}$ (6) dropped, while ${\text{E}}_{K}$ rose (5). The hypoxic-induced delayed depolarizations can be viewed as a sequence of events: (1) rapid reduction of O_2_ supply (hypoxia). (2) Na^+^-K^+^ pump current fell to 1% of its initial magnitude over 2.95 s as ATP was consumed (Fig 3E); (3) $↑{\text{V}}_{m}$ rose with loss of outward Na^+^-K^+^ pump current; then fell due to K^+^ channel responses to ${\text{V}}_{m}$. (4) $↑[K^{+}{]}_{o}$ due to Na^+^-K^+^ pump failure, (5) $↑{\text{E}}_{K}$, (6) $↓{\text{E}}_{Na}$, (7) reduced outward $I_{K}$ was less than (8) reduction in inward $I_{Na}$; (9) elevated ${\text{V}}_{m}$ to spike threshold, which produced only a partial AP due to the fall in ${\text{E}}_{Na}$. Despite inactivation of the Na^+^ channel shortly after opening (falling below 1% of peak conductance by 15 s), the neuron cannot repolarize due to the elevated ${\text{E}}_{K}$. (10) $↑[K^{+}{]}_{o}$ and further depolarization, produced by large and sustained $I_{K}$. (11) $↓[K^{+}{]}_{o}$ due to extracellular diffusion, while ${\text{V}}_{m}$ remained elevated as intracellular K^+^ continued to be replaced with Na^+^. Small high-frequency oscillations in the ${\text{V}}_{m}$ at the soma were produced by Na^+^ channel activity. Changes in extracellular concentration were smaller than intracellular changes, even with the currents scaled 5-fold (see Methods), as the volume of the modeled ECS exceeds that of the neuron. This is due to our modeling of a single neuron; in tissue the ECS would only be ~20% of the total volume (see Discussion). Simulations without an astrocyte, showed it only had minor effect of the state of the neuron via gradual clearance of extracellular K^+^ (S1 Text and S1 Fig).

During sustained hypoxia, synaptic mechanisms produced distinct somatic and dendritic responses (Fig 3F). The sequence of events that affected the somatic membrane potential produced similar disruptions in the dendrites: (3) $↑{\text{V}}_{m}$ was similar at the soma and basal dendrites, as Na^+^-K^+^ pump activity fell rapidly (to less than 1% at the soma and ~6% at the basal dendrites within 3 s) with many neighboring dendrites competing for limited O_2_. In contrast, the sparser apical dendrites were relatively spared, with little change in the Na^+^-K^+^ pump within 3 s, eventually reaching a plateau of 62%. (9) The range of membrane potentials also results from the residual Na^+^-K^+^ pump current, which was largest in the apical dendrites. (10) The partial AP triggered glutamate release, which activated synaptic mechanisms in the dendrites that were absent in the soma (particularly AMPAR). These synaptic mechanisms produced a larger and more rapid rise in dendritic ${\text{V}}_{m}$ than somatic ${\text{V}}_{m}$, despite similar extracellular environments for the soma and basal dendrites. (11) $↓[K^{+}{]}_{o}$ due to extracellular diffusion, and was far higher around the soma due to efflux from the soma and multiple neighboring dendrites. However, ${\text{V}}_{m}$ remains elevated due to the almost complete loss of $[K^{+}{]}_{i}$, with ${\text{E}}_{K}$ ~−10 mV. The synaptic currents that led to higher dendritic than somatic ${\text{V}}_{m}$ in Fig 3F, were dwarfed by greater changes caused by the large and rapid rise of ${\text{E}}_{K}$ in Fig 3C.

Sustained hypoxic effects were delayed by seconds rather than the millisecond shifts seen with high K^+^, reflecting the temporal buffering provided by the time required for ATP consumption and pump failure (compare Fig 3D–3F with Fig 3A–3C). However, depolarization, once it occurred, was considerably more pronounced under hypoxia as without ATP the pumps were unable to compensate. High extracellular K^+^ and hypoxia both led to the breakdown of ion homeostasis, but over different timescales and via distinct mechanisms. The rapid initial increase in extracellular K^+^ can be viewed as the loss of charge from one of the neuron’s batteries (${\text{E}}_{K}$), which led to a rapid initial disruption of ion homeostasis (initial 25 ms shown in Fig 3B). The loss is followed by a slower secondary rise in ${\text{E}}_{K}$ and a fall in ${\text{E}}_{Na}$  further reducing the neuron’s batteries as ${\text{V}}_{m}$ rises. In contrast, the rapid reduction in O_2_ produced slower initial changes in both Nernst potentials and ${\text{V}}_{m}$ (Fig 3E). After ~12 s, the partial AP led to the almost complete loss of ${\text{E}}_{K}$ and ${\text{E}}_{Na}$, producing much larger shifts in ${\text{V}}_{m}$. These differences between hypoxic and elevated $[K^{+}{]}_{o}$ conditions are partly due to the Na^+^-K^+^ pump, a current source that charges the neuron’s batteries. Hypoxia limited the capacity of the Na^+^-K^+^ pump and elevated K^+^ increased the drive; in both cases, the drive exceeded capacity. However, the residual Na^+^-K^+^ pump current at the soma was 9.9 fold greater under elevated K^+^ than when O_2_ was reduced.

Hypoxia and elevated K^+^ occur in HSD in the penumbra of ischemic stroke. Simulation of both insults together showed the initial dynamics are dominated by the effects of elevated K^+^, similar to Fig 3B. Over time, the membrane potentials were similar to those for hypoxic conditions (Fig 3F), resulting from the breakdown of ion homeostasis.

### 2.3. Ischemia and SD produced different distributions of ion concentration in the neuron

**Fig 4:**
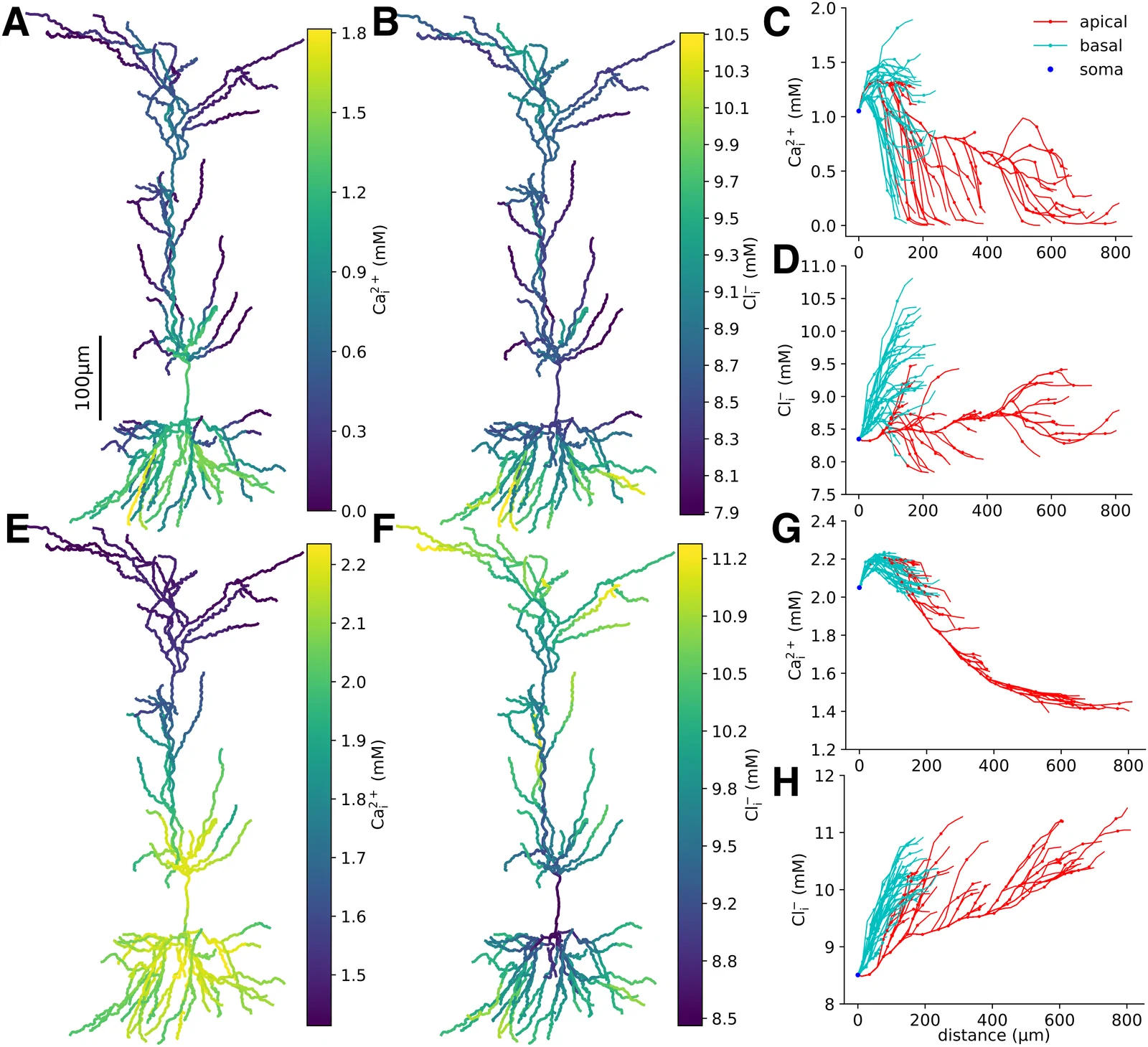
Elevated K^+^ and hypoxia produce different distributions of Ca^2+^ and Cl^-^. Both Ca^2+^ and Cl^-^ are most elevated in the basal dendrites under normoxic conditions with elevated K^+^ **(A-D)**. Under normoxic conditions with elevated K^+^: **(A)** ${\text{Ca}}_{cyt}^{2+}$ differed in soma (1.05 mM), basal dendrites (1.09 mM to 1.24 mM), and apical dendrites (1.21 mM to 0.20 mM). **(B)** ${\text{Cl}}_{cyt}^{-}$ increased with distance from the soma (8.35 mM) in the basal dendrites (8.32 mM to 9.90 mM), whereas apical dendrites have a lower concentration that does not depend on distance from the soma (8.32 mM to 8.79 mM). **(C)** ${\text{Ca}}_{cyt}^{2+}$ peaked in the basal dendrites (at 1.89 mM 167 μm from the soma) **(D)** ${\text{Cl}}_{cyt}^{-}$ also peaked with ${\text{Ca}}_{cyt}^{2+}$ in the basal dendrites (at 10.81 mM 167 μm from the soma). Hypoxia induced elevated Ca^2+^ in the basal dendrites, with greater accumulation of Cl^-^ in the distal apical dendrites **(E-H)**. Under hypoxic conditions: **(E)** ${\text{Ca}}_{cyt}^{2+}$ was most elevated at the soma (2.05 mM) and basal dendrites (2.07 mM to 2.01 mM), with similar concentrations in the proximal apical dendrites and lower concentrations in the distal apical dendrites (2.12 mM to 1.45 mM). **(F)** ${\text{Cl}}_{cyt}^{-}$ increased with distance from the soma (8.51 mM in both basal (8.54 mM to 10.37 mM) and apical dendrites (8.48 mM to 11.04 mM), **(G)** ${\text{Ca}}_{cyt}^{2+}$ peaked in the basal dendrites (2.24 mM at 74 μm from the soma) and declined in the apical dendrites with distance from the soma. **(H)** ${\text{Cl}}_{cyt}^{-}$ accumulation increased linearly with distance from the soma 1.61 mM/mm (R^2^ = 0.370), with greater rises in the basal 7.87 mM/mm (R^2^ = 0.591) than apical 1.93 mM/mm (R^2^ = 0.509) dendrites. Concentration were taken at 60 s after the rapid changes caused by depolarization. Concentration ranges in the dendrites were taken at the closest and furthest parts from the soma; apical dendrites (154 μm and 773 μm) and basal dendrites (35 μm and 203 μm).

Disruption of ion homeostasis caused highly variable Ca^2+^ accumulation across neuronal compartments, revealing compartment-specific vulnerabilities driven by local energy supply, transporter activity, and morphology (Fig 4). Elevated extracellular K^+^ (a model for SD) led to orders-of-magnitude increase in Ca^2+^ concentrations throughout, from 60 nM into the mM range (Fig 4A and 4C). The Ca^2+^ increase was greatest in the basal dendrites, with comparable values in some proximal apical dendrites, while distal apical dendrites showed less accumulation. Some dendritic compartments were spared significant rises in ${\text{Ca}}_{cyt}^{2+}$. The large range in ${\text{Ca}}_{cyt}^{2+}$ concentrations reflects a pattern of vulnerabilities in the neuron. Different patterns of NMDAR and Na^+^-Ca^2+^ exchange currents determined the differences in ${\text{Ca}}_{cyt}^{2+}$ in basal vs. apical dendrites. Tightly packed basal dendrites competed for limited O_2_ and suffered greater reduction in ATP than the distal apical dendrites. Residual Na^+^-K^+^ pump activity was lower in the basal dendrites, which led to higher $[{Na}^{+}{]}_{i}$, and thus limited Ca^2+^ clearance via the Na^+^-Ca^2+^ exchange (Fig 4A and 4C) Further reduction in Ca^2+^ elimination was driven by decreased ATP-limited Ca^2+^ pump and SERCA activity. NMDAR activity depended on accumulation of glutamate, while Na^+^-Ca^2+^ exchange increased with elevated $[{Na}^{+}{]}_{i}$ and ${\text{Ca}}_{cyt}^{2+}$. The rise in $[{Na}^{+}{]}_{i}$ was most pronounced in compartments where the Na^+^-K^+^ pump was overwhelmed and ATP declined. The role of both ATP and glutamate tied these changes to the morphology.

The rise of ${\text{Cl}}_{cyt}^{-}$ was less marked, increasing up to 64% from a baseline of 6.6 mM (Fig 4B and 4D). The pattern of Cl^-^ accumulation under elevated K^+^, was similar to that of ${\text{Ca}}_{cyt}^{2+}$. Concentrations of Cl^-^ were higher in the basal than the apical dendrites. The reversal of KCC2 drove the Cl^-^ increase as the dominant Cl^-^ current. KCC2 normally utilizes the K^+^ gradient to remove Cl^-^ from the neuron, but may reverse direction under pathological conditions. KCC2 reversal occurred almost immediately, due to the shift in ${\text{E}}_{K}$ that resulting from elevated extracellular K^+^ (Fig 3B). In parts of dendrites KCC2 recovered, as there was sufficient ATP to maintain some Na^+^-K^+^ pump activity, increasing $[K^{+}{]}_{i}$ and restoring ${\text{E}}_{K}$ above ${\text{E}}_{Cl}$.

Under hypoxic conditions, the rise in ${\text{Ca}}_{cyt}^{2+}$ was more pronounced with greater differences between proximal and distal dendrites (Fig 4E and 4G). As with the normoxic case, the rise in ${\text{Ca}}_{cyt}^{2+}$ depended on the balance between NMDAR and Na^+^-Ca^2+^ exchange, which tie Ca^2+^ concentrations to glutamate and O_2_, which are affected by the dense dendritic arbor near the soma.

The distribution of Cl^-^ was well-described by path distance from the soma, reaching its maximum concentration in apical dendrites. Concentrations of Cl^-^ rose with distance from the soma at an average of 1.61 mM/mm in our simulation, with more pronounced increases with distance in the basal than in the apical dendrites. Unlike under elevated K^+^, under hypoxic conditions KCC2 reversed throughout the neuron, with differences in Cl^-^ caused by the timing and extent of the reversal. KCC2 reversal was delayed as the changes in ${\text{E}}_{K}$ were slower under hypoxic conditions (Fig 3E). KCC2 reversal occurred first in a basal dendrite before the apical dendrites. The greater increase in ${\text{Cl}}_{cyt}^{-}$ in the apical dendrites resulted from the breakdown of the ${\text{E}}_{K}$ and ${\text{E}}_{Na}$, which were both lower on average in the apical dendrites. KCC2 is driven by the difference between ${\text{E}}_{K}$ and ${\text{E}}_{Cl}$, and NKCC1 (a sodium-potassium-chloride transporter) by the difference between both ${\text{E}}_{K}$ and ${\text{E}}_{Na}$ with ${\text{E}}_{Cl}$.

**Fig 5:**
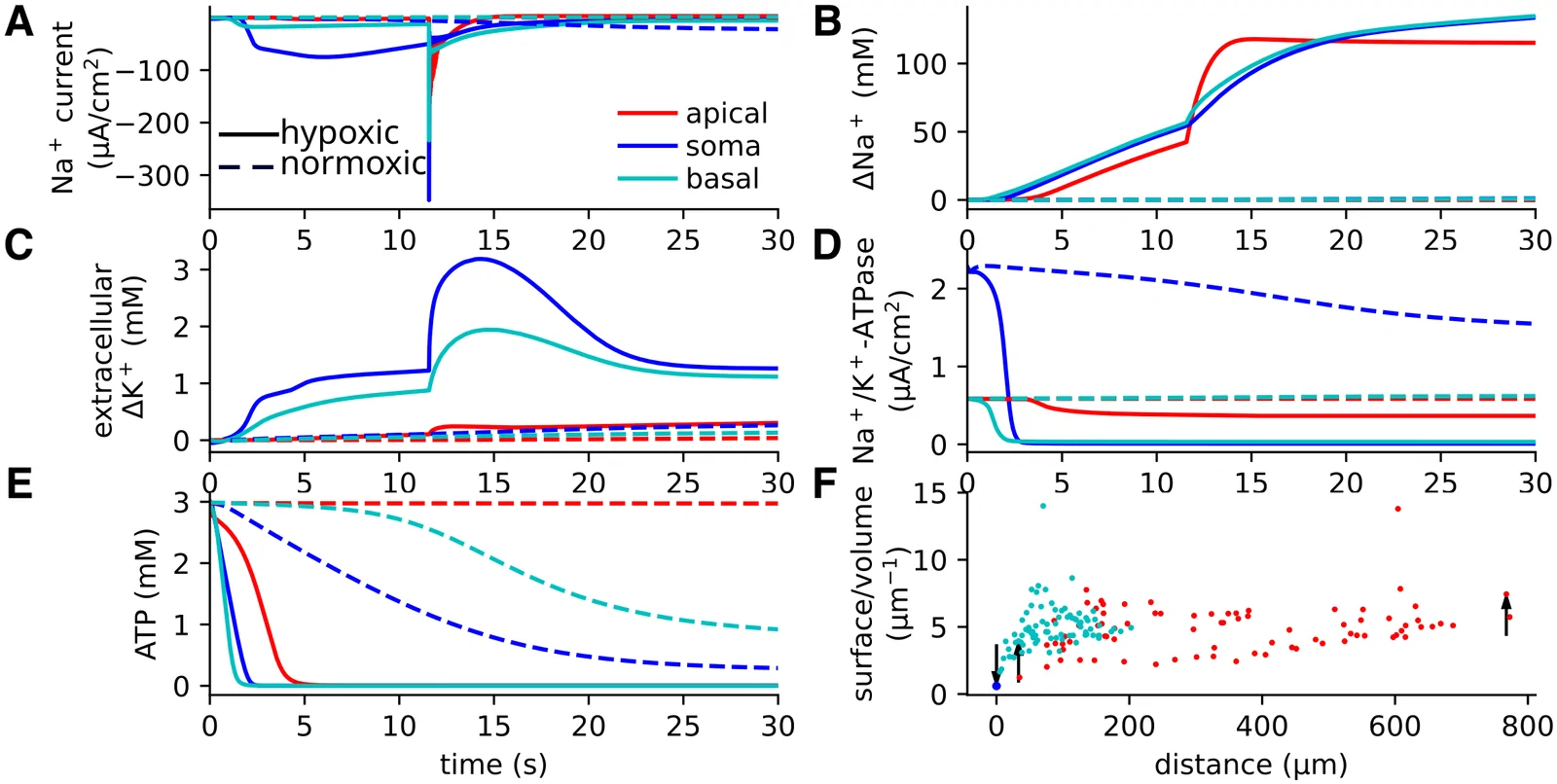
The dense dendritic arbor around the soma leads to greater vulnerability to hypoxia in the basal dendrites. **(A)** In normoxic conditions, there is little change in Na^+^ currents. Under hypoxic conditions, however, there were substantial sustained inward Na^+^ currents. **(B)** Hypoxia caused a rise in $[{Na}^{+}{]}_{i}$ from baseline throughout the neuron. Prior to depolarization (~12 s), the rise is most rapid in the basal dendrites and soma; following depolarization, the rise is greatest in the apical dendrites. $[{Na}^{+}{]}_{i}$ then plateaus in the apical dendrites, leading to higher concentration in the soma and basal dendrites. **(C)** Major increase in extracellular K^+^ in the soma and basal dendrites due to the large dendritic arbor near the soma. There is only a small rise at the apical dendrites. **(D)** The Na^+^-K^+^ pump current declined in the soma (falling to 10% at 2.39 s) and basal dendrites (10% at 2.07 s) under hypoxia due to the consumption of ATP. The pump was spared in apical dendrites, remaining at 62.4% by 60 s. **(E)** ATP was more rapidly consumed in the basal dendrites due to both earlier onset of Na^+^-K^+^ pump drive (due to Na^+^ influx) and larger current (due to extracellular K^+^), so they depolarized before the rest of the neuron. While ATP was consumed in the normoxic condition, the difference did not substantially impact the action of the Na^+^-K^+^ pump. **(F)** The surface/volume ratio in the apical and basal dendrites are similar, but the distance from the soma and the dense basal dendritic arbor play a role in determining the rise in extracellular K^+^. Arrows indicate the parts of the neuron compared above. All Δs shown are relative to baseline.

Changes in Cl^-^ concentration distribution were driven primarily by the non-uniform disruption to K^+^ and Na^+^. Basal dendrites near the soma experience higher $[K^{+}{]}_{o}$ than apical dendrites (Fig 5C). As a result, ${\text{E}}_{K}$ was higher near the soma, so there was greater KCC2 activity and consequently less elevated ${\text{Cl}}_{cyt}^{-}$.

While the spatial distribution of ${\text{Cl}}_{cyt}^{-}$ dynamics predicts greater ${\text{Ca}}_{cyt}^{2+}$ concentrations in the apical dendrites, the osmotic pressure is greater at the soma (S1 Text and S3 Fig). However neurons lack aquaporins and water influx has been shown to depend on Cl^-^ transporter [13], so the ${\text{Cl}}_{cyt}^{-}$ distribution may confer higher susceptibility to beading morphology.

In both cases, energy-dependent clearance mechanisms could better respond to demand in apical compared to basal dendrites. Glutamate-dependent NMDAR activation significantly increased Ca^2+^ accumulation and altered depolarization dynamics. We identified the contribution of glutamate-NMDAR influx by assessing a model without NMDAR. Under hypoxic conditions, this model produced a similar distribution of Ca^2+^ and Cl^-^ with lower overall intracellular concentrations. In the absence of glutamate, elevated K^+^ led to large high frequency oscillations rather than sustained depolarizations and resulted in a far smaller rise in ${\text{Ca}}_{cyt}^{2+}$.

### 2.4. Hypoxia contributes to pathological changes of calcium and chloride throughout the cell

**Fig 6:**
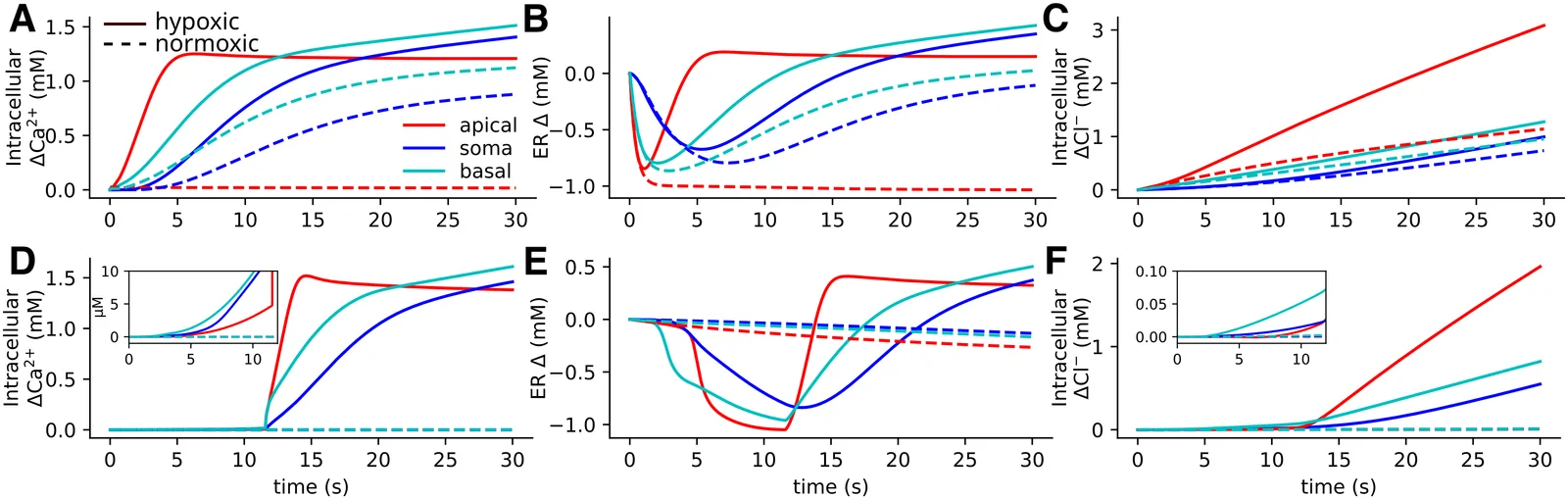
Elevated K^+^ led to a rapid rise in both the Ca^2+^ and Cl^-^ concentrations, while hypoxic changes are delayed. Rapid concentration changes followed neuronal depolarization that occurred almost immediately (~25 ms) with elevated K^+^ **(A-C)**, or (~12 s) with baseline K^+^ **(D-F)**. **(A)** Under hypoxic conditions (solid lines), the rise in ${\text{Ca}}_{cyt}^{2+}$ was most pronounced in the apical dendrites, whereas under normoxic conditions, the apical dendrites were spared. **(B)** Ca^2+^ in the ER showed a decline followed by recovery as it equilibrates to the elevated intracellular Ca^2+^. **(C)** Cl^-^ concentration rose gradually in the soma and basal dendrites and had the greatest increase in the apical dendrites under both hypoxic and normoxic conditions. In the absence of applied K^+^ (D-F) there was little change in concentrations under normoxic conditions (dashed-line), with hypoxia leading to substantial changes following depolarization at ~12 **s. (D)** The smaller initial increase in ${\text{Ca}}_{cyt}^{2+}$ were due to influx from the ER and brief reversal of Na^+^-Ca^2+^ exchange (between 3.65 s and 8.64 s at the soma). The rise in ${\text{Ca}}_{cyt}^{2+}$ following the AP was initially greatest in the apical dendrites, which then plateaued. Whole basal and somatic ${\text{Ca}}_{cyt}^{2+}$ rose more slowly; they ultimately exceeded apical concentrations. **(E)** The decline in ER Ca^2+^ preceded the depolarization, which reversed following depolarization as ER Ca^2+^ rose to equilibrate with cytosolic concentrations. **(F)** After an initially rapid rise in the basal dendrites (inset), the long-term rise in ${\text{Cl}}_{cyt}^{-}$ was greatest in the apical dendrites. All Δs shown are relative to baseline.

Elevated extracellular K^+^ led to immediate rises in Ca^2+^ and Cl^-^ (Fig 6A–6C), that varied with location and available O_2_. Under hypoxic conditions, ${\text{Ca}}_{cyt}^{2+}$ rose most rapidly in the apical dendrites before it plateaued and was surpassed by the basal and somatic concentration (Fig 6A solid lines). Under normoxic conditions, elevated $[K^{+}{]}_{o}$ produced substantial rises in ${\text{Ca}}_{cyt}^{2+}$ at the basal dendrites and soma. In contrast, apical dendrites were able to maintain lower ${\text{Ca}}_{cyt}^{2+}$ due to less competition for O_2_ and ATP (Fig 6A dashed lines). The ER Ca^2+^ initially declined, but then reversed and recovered as it reached equilibrium with the elevated cytosolic concentration (Fig 6B). Again, apical dendrites were spared in normoxic conditions. The rise in Cl^-^ was most pronounced in the apical dendrites, for both normoxic and hypoxic conditions (Fig 6C). There was less competition for available O_2_ in apical dendrites compared with the densely packed basal dendrites, which led to higher $[K^{+}{]}_{i}$ and lower ${\text{E}}_{K}$, leading to lower KCC2 Cl^-^ influx or recover KCC2 Cl^-^ efflux in some dendrites under normoxic conditions.

With baseline K^+^ under hypoxic conditions, disruption of the balance between K^+^ and Na^+^ produced pathological changes in ${\text{Ca}}_{cyt}^{2+}$ and ${\text{Cl}}_{cyt}^{-}$ (Fig 6D–6F). The majority of the pathological accumulation of Ca^2+^ and Cl^-^ occurs with depolarization at ~12 s. Prior to depolarization, cytosolic Ca^2+^ increased gradually throughout the neuron by about two orders of magnitude, from 60 nM to μm concentrations. These ${\text{Ca}}_{cyt}^{2+}$ dynamics were driven by the ER store, Na^+^-Ca^2+^ exchange, and buffering. Somatic Ca^2+^ rose substantially pre-depolarization (Fig 6D inset), aided in part by a brief reversal of the Na^+^-Ca^2+^ exchange. Basal dendrites showed a comparable rise, while the apical concentration rose about a third as much. The Na^+^-Ca^2+^ exchange clearance was reduced and only briefly reversed in parts of the dendrite and at the soma. In all regions, ER Ca^2+^ was largely depleted during the pre-depolarization phase (Fig 6E solid line). This decrease exceeds the increase in cytosolic Ca^2+^ even after adjusting for differences in volumes, likely due to cytosolic Ca^2+^ buffering and residual clearance via Na^+^-Ca^2+^ exchange and Ca^2+^ pump.

Depolarization at ~12 s triggered a rapid rise in ${\text{Ca}}_{cyt}^{2+}$ driven by NMDAR and modulated by Na^+^-Ca^2+^ exchange, leading to compartment specific dynamics Ca^2+^ changes (Fig 6D solid line). Dendritic ${\text{Ca}}_{cyt}^{2+}$ increased by almost two orders of magnitude in less than two seconds. Apical Ca^2+^ rose most rapidly, followed by basal Ca^2+^, however, apical concentrations plateaued. Dendritic concentrations were driven by the balance between NMDAR Ca^2+^ influx and Na^+^-Ca^2+^ exchange efflux. After the rapid rise, the Na^+^-Ca^2+^ exchange became sufficiently balanced in the apical dendrites that the Ca^2+^ pump was able to reduce the concentration. Somatic Ca^2+^ again increased with brief reversal of Na^+^-Ca^2+^ exchange but was primarily driven by influx from the dendrites, trailing the basal concentrations. With the cytosolic Ca^2+^ elevated, the ER concentrations in all regions recovered, then exceeded baseline as they moved towards equilibrium with the elevated cytosolic concentrations (Fig 6E solid line).

${\text{Cl}}_{cyt}^{-}$ likewise exhibited a gradual increase prior to depolarization and a significant rise thereafter, primarily driven by alterations in KCC2 and NKCC1 currents, revealing differential regulation across somatic, basal, and apical regions. Prior to depolarization, ${\text{Cl}}_{cyt}^{-}$ gradually increased, due to reductions in KCC2 current (Fig 6F inset). The largest increase in ${\text{Cl}}_{cyt}^{-}$ followed depolarization, driven primarily by the rise reversal of KCC2. (Fig 6F solid line). Apical ${\text{Cl}}_{cyt}^{-}$ showed larger and more rapid rises due to the distribution of $[K^{+}{]}_{i}$ and $[{Na}^{+}{]}_{i}$. The shifts in ${\text{E}}_{K}$ and ${\text{E}}_{Na}$ were more abrupt near the soma due to the greater reductions in O_2_, ATP production, and Na^+^-K^+^ pump activity.

Under normoxic and baseline $[K^{+}{]}_{o}$ (control) conditions, ${\text{Ca}}_{cyt}^{2+}$ or ${\text{Cl}}_{cyt}^{-}$ remained stable (Fig 6D and 6F dashed line). However, there was a gradual decline in ER Ca^2+^ concentration (Fig 6E dashed line).

### 2.5. Basal dendrites show greatest vulnerability to concentration changes

To understand the particular vulnerability of dendrites [9], we further explored differences in voltages and fluxes from soma to both apical and basilar dendrites. Here we compared hypoxic and normoxic conditions with baseline $[K^{+}{]}_{o}$ (Fig 5). The activity of Na^+^-K^+^ pump is crucial for maintaining ion homeostasis; its failure under ischemic conditions gives rise to specific dendritic vulnerabilities due to neuronal morphology. Na^+^-K^+^ pump activity was driven by excess $[{Na}^{+}{]}_{i}$, $[K^{+}{]}_{o}$ and available ATP. Inward somatic Na^+^ currents per unit area were far larger than dendritic currents, but the resulting substantial and rapid increase in $[{Na}^{+}{]}_{i}$ was similar in the dendrites due to the greater surface/volume ratio (Fig 5A and 5B). While the increase in $[{Na}^{+}{]}_{i}$ was similar throughout the neuron, the rise in $[K^{+}{]}_{o}$ was more prominent around the soma and basal dendrites (Fig 5C). The extracellular K^+^ did not saturate the Na^+^-K^+^ pump and so determined the maximum drive, reaching 50% of maximum first at the basal dendrite, then at the soma, while only reaching 62% of maximum in the apical dendrite. Na^+^ influx was driven by a feedback loop, where efflux declined due to a reduction in Na^+^-K^+^ pump from the loss of ATP, and the influx increased principally via the leak currents due to shifts in the Nernst potentials.

The Na^+^-K^+^ pump activity was determined both by demand ($[{Na}^{+}{]}_{i}$, $[K^{+}{]}_{o}$) and the supply of O_2_, leading to regional differences in activity and ion concentrations. There was a marked decline in Na^+^-K^+^ pump activity at the soma and basal dendrites, with apical dendrites spared (Fig 5D). The Na^+^-K^+^ pump currents depended on both the drive and ATP production, which in turn depended on available O_2_. Hypoxia led to ATP loss throughout the cell, but was most pronounced in the soma then the basal dendrite, while the apical dendrites were relatively spared (Fig 5E). Despite substantial ATP loss in the apical Na^+^-K^+^ pump, there was a large residual current in the apical dendrites. This Michaelis-Menten formulation of Na^+^-K^+^ pump ATP-dependence gave a ~10k fold reduction in Na^+^-K^+^ pump current. This reduction was counterbalanced with increased demand (elevated $[{Na}^{+}{]}_{i}$ and $[K^{+}{]}_{o}$), which in the absence of ATP constrains would give a ~100 fold increase in Na^+^-K^+^ pump activity. Currents in the neuron depend both on the mechanism and the area of the membrane, while concentrations depend on the volume of the compartment. We consider the surface/volume ratio of each compartment (Fig 5F). The compartment sizes were similar for basal and apical dendrites, with a difference driven by the density of compartments. The greater and more rapid rise in extracellular K^+^ and fall in O_2_ in the basal dendrite was due to the dense dendritic arbor and close proximity to the soma. This gave rise to the larger Na^+^-K^+^ pump currents in the apical dendrites.

### 2.6. Oxygen dependence of ischemic depolarization is all-or-none; concentration changes are gradual

**Fig 7:**
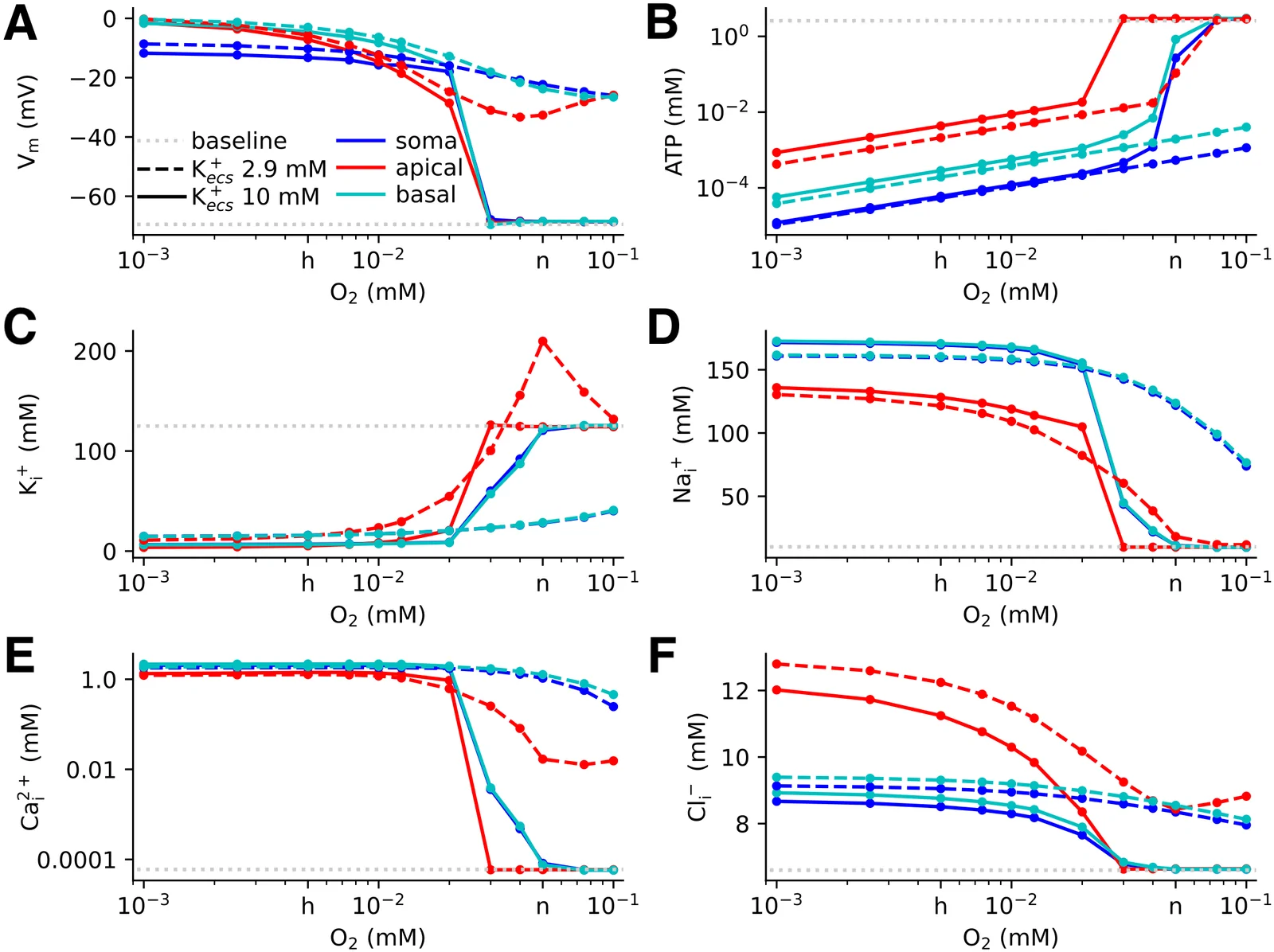
All-or-none depolarizations occurred when O_2_ was limited or extracellular K^+^ was elevated. **Concentration changes depended on the extent and nature of the stressor.** With elevated extracellular K^+^ (10 mM) depolarization occurred rapidly (within 30 ms) at all O_2_ levels considered (10^−3^ to 0.1 mM). In the absence of elevated K^+^ (2.9 mM) at and just below normoxic (‘n’ on the *x*-axis) O_2_ levels, the neuron maintained RMP; at lower or hypoxic (‘h’) levels, depolarization occurred after a delay (up to 30 **s)**. This figure shows the relation between the limited O_2_ supply and: **(A)** Membrane potential, **(B)** ATP, **(C)** K^+^, **(D)** Na^+^, **(E)** ${\text{Ca}}_{cyt}^{2+}$, **(F)** ${\text{Cl}}_{cyt}^{-}$.

With baseline K^+^ concentrations, there was an abrupt transition in membrane potential between oxygen levels that could and could not maintain the RMP, consistent with an all-or-none SD process (Fig 7A dashed-lines). Depolarization occurred earlier at lower oxygen levels, suggesting faster SD propagation with more severe oxygen deprivation. Depolarization was abrupt, but intermediate states persisted without such large pathological changes in Ca^2+^ and Cl^-^ concentrations. Electrical stimulus produced a relatively abrupt transition between oxygen levels that could maintain the RMP (0.03 mM or more) and those that could not (0.02 mM or less). Pathological depolarization occurred between 9.5 s for 1 μm O_2_ and 28.3 s for 0.02 mM O_2_. Similar abrupt transitions occur with ATP, K^+^, and Na^+^ concentrations (Fig 7B–7D dashed-lines). The residual ATP was greatest in the apical dendrites (Fig 7B); this was reflected in higher $[K^{+}{]}_{i}$ and lower $[{Na}^{+}{]}_{i}$ due to residual Na^+^-K^+^ pump activity (Fig 7C and 7D). This in turn led to the greater accumulation in ${\text{Ca}}_{cyt}^{2+}$ in the basal dendrites (primarily due to $[{Na}^{+}{]}_{i}$ dependent Na^+^-Ca^2+^ exchange) and greater ${\text{Cl}}_{cyt}^{-}$ in the apical dendrites (due to ${\text{E}}_{K}$ dependent KCC2). With 0.03 mM O_2_ (while the neuron maintained RMP), there was a substantial reduction in ATP and subsequent partial loss of $[K^{+}{]}_{i}$ and accumulation of $[{Na}^{+}{]}_{i}$. These intermediate states persisted and led to a less dramatic increase in ${\text{Ca}}_{cyt}^{2+}$ and did not produce secondary pathological changes in ${\text{Cl}}_{cyt}^{-}$ (Fig 7E and 7F).

While the dynamics were different, the pathological response to hypoxia tended to a similar state for both elevated and baseline K^+^, with substantial difference only at higher O_2_ levels (Fig 7). Elevated K^+^ produced rapid depolarizations under all O_2_ levels (Fig 7A solid-lines). With sufficient O_2_ (above 0.03 mM), apical $[K^{+}{]}_{i}$ increased above baseline due to large sustained Na^+^-K^+^ pump currents. Accumulation peaked at 0.005 mM O_2_, due to the effects of Na^+^ channel activation leading to higher $[{Na}^{+}{]}_{i}$ and greater Na^+^-K^+^ pump drive (Fig 7D solid-lines). Depolarization caused by extracellular K^+^ led to Ca^2+^ accumulation, which primarily depends on the glutamate mediated NMDAR influx and Na^+^-Ca^2+^ exchange clearance, which was greatest in the basal dendrites (Fig 7E solid-lines). The accumulation of ${\text{Cl}}_{cyt}^{-}$ was a direct result of the shift in ${\text{E}}_{K}$ via KCC2 reversal, which was greatest in the apical dendrites (Fig 7F solid-lines).

## 3. Discussion

Motivated by histologic abnormalities associated with ischemic brain tissue, our simulation of a neuron under hypoxic vs. normoxic conditions gave insights into the mechanisms underlying cellular and subcellular responses to energy deficit or ionic overload. Such a simulation has implications for these effects at the level of bulk tissue and of neuronal networks.

Varicosities distributed along neurites (‘beading’) have been observed in a variety of neuropathologic conditions, including trauma [28], epilepsy [29], neurodegeneration, and ischemia [30,31]. Specifically, in neurons in the penumbra of an experimentally induced infarct in a mouse, dendritic beading morphology was temporally correlated with SD, with recovery between episodes until a terminal injury resulting in irreversible morphologic change [4]. Our results provide a mechanism by which SD links the osmotic imbalances to the beaded morphology in observed in ischemic lesions.

The model predicted three stages of response to hypoxic loss of homeostasis: (1) replacement of $[K^{+}{]}_{i}$ with $[{Na}^{+}{]}_{i}$ with little initial effect on membrane potential; (2) rapid depolarization of neurons due to opening of the Na^+^ channels, particularly in dendrites with the greatest ion concentration change; and (3) persistent ion concentration breakdown. By comparison, elevated $[K^{+}{]}_{o}$ without hypoxia led to (1) rapid depolarization due to the change ${\text{E}}_{K}$; (2) the shift in Nernst potential dwarfed the synaptic mechanisms; (3) leading to different distributions of ${\text{V}}_{m}$ and ions in the dendrites. The different consequences depending on the specifics of the relative severity of ionic and hypoxic insult suggest different neuroprotective therapies based on the pattern of pathophysiological patterns.

Our model demonstrated the influence of neuronal morphology on dendritic stressors due to regional differences in extracellular accumulations and local O_2_ consumption. Specifically, the density of the basal dendritic arbor near the soma is a region of high vulnerability due to (1) reduced clearance of glutamate with activation of NMDAR leading to Ca^2+^ influx; (2) greater $[K^{+}{]}_{i}$ and $[{Na}^{+}{]}_{i}$ due to higher $[K^{+}{]}_{o}$; (3) greater local competition for O_2_. We predict that high Ca^2+^ in basilar dendrites would produce excitotoxicity with earlier loss of these dendrites. Elevated ${\text{Ca}}_{cyt}^{2+}$ does not directly cause apoptosis or dendrite loss. However, it is associated with loss of microtubule-associated protein 2 from dendrites [15]. Elevated ${\text{Ca}}_{cyt}^{2+}$ occurs in both the early and late stages of the apoptotic pathway [32]. Such pathways are beyond the scope of the current model.

By contrast, hypoxic disruption of ${\text{E}}_{K}$ and ${\text{E}}_{Na}$ resulted in greater ${\text{Cl}}_{cyt}^{-}$ in the apical dendrites due to KCC2 imbalance. Since acute dendritic beading represents increased osmotic pressure, manifested as focal swellings due to the heterogeneous distribution of ion channels and cytoskeletal anchors [17], we predict that this would produce greater dendritic beading in apical as compared to basilar dendrites. Osmotic pressure may be greater near the soma (S1 Text and S3 Fig), but dendritic beading is not a simple osmotic event; it depends on Cl^-^ and its co-transporters [13]. This asymmetric localization of damage will have differential cognitive consequences due to the importance of apical dendrites in higher-level feedback, with predominance of basilar dendrites in local activity spread.

Our study has added a focus on extracellular transmission, ion homeostasis, and a more detailed model of calcium compartmentalization and dynamics compared to prior studies. While our work is a substantial simplification of the multiscale interactions of different cells and pathways that are known to occur in brain tissue, it is also more complex and detailed than prior models. Prior modeling work utilized simplified morphologies and focused on conditions for SD initiation, neuron-glia interactions, and their contribution to seizure-like activity [23,24,33]. These models featured electroneutrality and volume dynamics, maintained by balancing changing anion concentrations with shifts in Cl^-^ and changes in osmolarity, using extracellular K^+^ alone in SD initiation, with greater astrocyte buffering delaying or preventing SD. Unlike the model presented here, these studies only considered lateral diffusion in a thin layer surrounding the cell membrane, so there was no influence from the dense dendrite arbor near the soma.

Like all models, ours is necessarily an approximation of reality. The model presented here does not include electroneutrality or volume dynamics; instead, Cl^-^ was modeled independently with specific mechanisms responsible for its regulation. Experimental observations on the speed of dysregulation following oxygen glucose deprivation are on the order of minutes to hours [34,35]. The more rapid breakdown shown in our model is likely due to the simplifications inherent in our metabolic model. Biologically, multiple mechanisms are employed to maintain ATP levels under oxygen deprivation, including glycolysis, lactate production, and mitochondrial adaptations. Anaerobic glycolysis, utilizing lactate provided by glia via the neuron lactate shuttle, offers an additional supply of ATP despite reduced O_2_ [36]. This lactate shuttle may be important for the post-ischemic survival of neurons [37]. While we did not explicitly consider any of these mechanisms as additional sources of ATP, they would serve to shift the O_2_ dependency curve to right (Fig 7).

Furthermore, unlike in our model, glycolytic ATP production is not uniformly distributed in the cytoplasm but highly enriched in the dendritic spines and the postsynaptic density [38]. There are more synapses on the apical than basal dendrites [39], but the apical dendrites are also larger. For our model, this gives a slightly lower density of synapses in the apical (1 per 1.38 μm^2^) than in the basal (1 per 1.12 μm^2^). This variation in anaerobic ATP production might reduce the distance-dependent difference seen between compartments in our elevated K^+^ simulation. Under ischemic conditions, both O_2_ and glucose would likely be exhausted.

In this study we focused on a single cell, but each cell is embedded in tissue, the neuropil, so that extracellular fluid would be strongly influenced by other cells nearby, which will be experiencing SD as well. This could change the observed differential effects between apical and basal dendrites, depending on the degree of alignment of dendritic elements, far more aligned in hippocampus than in areas of neocortex. The length of the CA1 dendritic arbor is greatest in the stratum oriens (OR) (47±7%), where the basal dendrites of the CA1 pyramidal neuron are located [40]. By contrast, less of the arbor is comprised of apical dendrites in the stratum radiatum (RAD) (35±9%) and stratum lacunosum-moleculare (LM) (19±3%). Our model captures this as the single CA1 neuron gives rise to localized O_2_ sink and K^+^ source in the dense basal dendritc arbor. The total density of dendrites in the neuropil is similar between layers OR (0.33±0.07), RAD (0.40±0.11), and LM (0.35±0.09) [41], so neighboring neurons are unlikely to preferentially consume O_2_ or release K^+^. At the nanoscale, quantum dots diffusion trajectories exhibit the greatest confinement in the OR [42], which could support the feedback-loop we identified. The O_2_ concentration at rest and blood vessel responsiveness are similar through the CA1 [43], suggesting there is not additional supply in the OR to accommodate the increased demand of the basal dendrites. Hypoxic simulations with elevated $[K^{+}{]}_{o}$, both with and without glutamate release, also produced the same distance-dependent relationships, with peak ${\text{Ca}}_{cyt}^{2+}$ in the basal dendrites ${\text{Cl}}_{cyt}^{-}$ in distal-apical dendrites. The persistence of these patterns in a model of SD suggests feedback, and the cell morphology may still produce these ion gradients even when the extracellular concentrations are perturbed by its neighbors.

In stroke-like conditions, Weilinger et al. showed that dendritic beading is associated with high intracellular ${\text{Cl}}_{cyt}^{-}$ [44]. Our model’s prediction of greater ${\text{Cl}}_{cyt}^{-}$ at greater distances from the soma is thus consistent with experimental findings that show beading occurs first distally and later appears proximally [16]. ${\text{Cl}}_{cyt}^{-}$ imaging in living animals is challenging, and we are unaware of more direct experimental evidence for the distribution of ${\text{Cl}}_{cyt}^{-}$ following stroke onset.

Ion concentration changes during ischemia are not driven solely by a breakdown in the homeostatic mechanisms modeled here; for example, in ischemia, a rapid influx of CSF brings additional Na^+^, contributing to cytotoxic edema [45]. Neurovascular coupling is a key component of the dynamics of SD pathology. In healthy tissue, SD can lead to a normal hemodynamic response. In this regime, the increase in metabolic consumption of O_2_ exceeds the increase in blood flow (hyperemia), so there is a decline in ATP. The hyperemia outlasts SD and is followed by a prolonged decline in blood flow (spreading oligemia). In pathological conditions, SD can produce an inverse hemodynamic response, where elevated K^+^ and reduced NO inhibit release of vasodilators and augment vasoconstriction [3]. The neurovascular response in SD has been widely studied in vitro [13,46,47] where O_2_ and glucose levels are controlled by bath application. Mathematical and computational approaches have also been used to study SD [21,23,24,33,48], where O_2_ or its effects are model parameters.

Intracellular Ca^2+^ accumulation in our model was driven primarily by NMDAR-mediated influx. When glutamate was removed from simulations, so there was no NMDAR activity, ${\text{Ca}}_{cyt}^{2+}$ was still elevated due to the effects of Na^+^-Ca^2+^ exchange. Experiments have shown that extracellular Ca^2+^ falls to ~10% of control during ischemia, with most of the uptake attributed to neurons, with estimated rises in ${\text{Ca}}_{cyt}^{2+}$ on the order of 100 nM [49,50]. Our model predicted excessive ${\text{Ca}}_{cyt}^{2+}$ changes relative to experimental observations; this excess was due to modeling a single cell; in reality, the 0.028 mm^3^ space modeled would be occupied by ~2,500 cells, sharing available extracellular Ca^2+^ between them.

Reducing the available extracellular Ca^2+^ in the model produced limited ${\text{Ca}}_{cyt}^{2+}$ increases, consistent with experimental estimates. These low-Ca^2+^ simulations did not affect the distance-dependent patterns or the mechanisms that gave rise to them (S1 Text; S2 Fig).

Additional unmodeled aspects of cellular morphology are anticipated to be consistent with the direction of changes observed in the model. Mitochondria occupy a greater fraction of intracellular volume in the basal and oblique dendrites than in the rest of the cell [51], but as the limiting factor of ATP production during ischemia is O_2_, the distribution of mitochondria would not negate the dendritic vulnerability predicted in this model. Cellular swelling due to osmotic pressure would likely dilute intracellular concentrations while reducing extracellular volume, leading to increased extracellular concentrations.

The model described here offers, to our knowledge, the most biophysically detailed model to date of the combined effects of ionic imbalance and ischemia at the subcellular level. This model will be used in future studies considering recovery from SD and coupling these features in network simulations. This offers the potential for new insights into cellular response to a variety of insults including primary ischemic, migraine, inflammatory, traumatic and neoplastic, with new opportunities for exploring the effects of neuroprotective intervention to prevent damage beyond the primary site of injury.

## 4. Materials and methods

**Fig 8:**
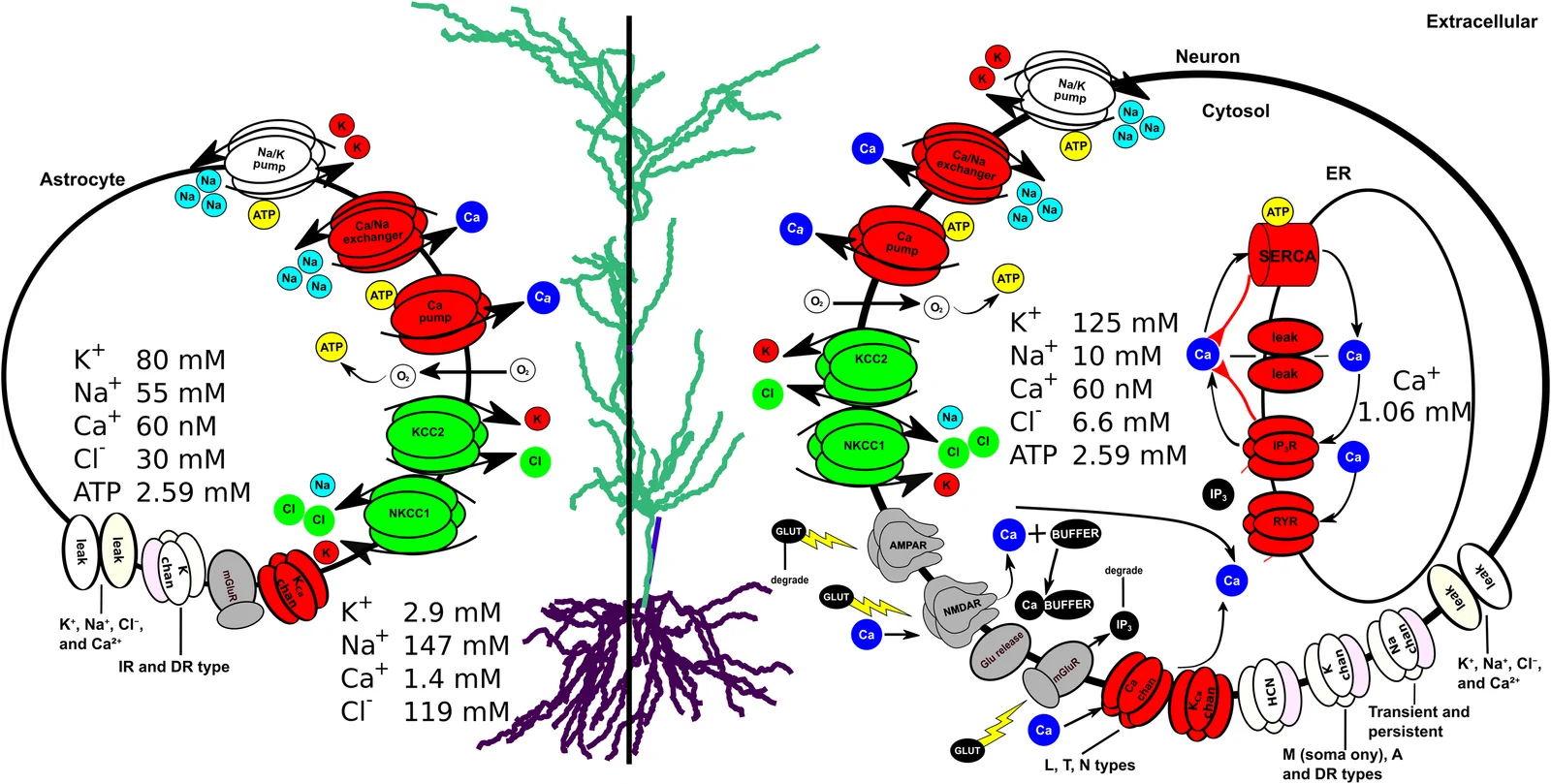
Diagram showing the mechanisms and cells in our model. Astrocyte mechanisms are on the left; neuron mechanisms are on the right. Default baseline concentrations are shown for each cell type. The astrocyte morpholgy is modeled as a simple cylinder next to the neuron, while the neuron uses a reconstructed morphology taken from the base model. The different compartments are: the astrocyte (black), basal dendrites (dark blue), apical dendrites (green), and axon segment (blue). Neuronal synaptic mechanisms (AMPAR, NMDAR, Glutamate release, mGluR) are only present in the dendrites.

We based our study on a prior hippocampal CA1 pyramidal neuron model, extensively studied in ischemia and SD [49,52,53], since large pyramidal neurons are particularly vulnerable to metabolic challenges [54](ModelDB: 244688) [18]. The dendrites and soma have several K^+^ channels: delayed rectifying, A-type, Ca^2+^-activated and Ca^2+^-dependent, as well as $I_{h}$. The axon and soma, in addition, have a non-inactivating K^+^ current (M-current). Both the soma and dendrites have N-, L-, and T-type Ca^2+^ channels. There are Na^+^ channels in all parts of the neuron, although the dynamics (inactivation) differ. The model morphologies and mechanisms together with the baseline concentrations are shown in Fig 8. Diffusion closely coupled the two intracellular shells with radial diffusion between them: 0.85 *r* (where *r* is the radius) to plasma membrane representing surface concentrations, $[X{]}_{i}$; center to 0.85 *r*, further subdivided as cytosol $[X{]}_{cyt}$: 83% of the volume; endoplasmic reticulum ER; $[X{]}_{ER}$, 17% of volume; $[X{]}_{o}$: extracellular.

The model includes both intracellular and extracellular reaction-diffusion, using the NEURON simulator [55,56] including its reaction-diffusion module, which supports coarse-grained volume-averaged models of the extracellular space [57,58].

### 4.1. Energy and metabolism

We explicitly modeled oxygen and ATP concentrations: Oxygen is consumed to produce ATP and restored at a rate proportional to how depleted it is relative to a baseline concentration for normoxic or ischemic/hypoxic tissue [21].

We used a simplified ATP model [22], where ATP returns to a set steady-state (ATP_*ss*_) of 2.59 mM [59] with time constant 3.9 ms. This process depends on the amount of O_2_ available, with 6 O_2_ molecules capable of producing 30 molecules of ATP [60]. The model includes rapid intracellular diffusion of ATP, and both intracellular and extracellular diffusion of O_2_ [61,62]. For simplicity, rates of the energy-dependent Na^+^-K^+^ pump [63], SERCA [64], and the plasma membrane calcium pump (PMCA) were modified to include Michaelis-Menten kinetics for their dependence on ATP, while keeping the same maximum rate.

### 4.2. Glutamate and synapse models

Our homeostatic glutamate model was based on [20], where each segment is equipped with an “average-synaptic cleft” and glutamate release is based on a threshold of the membrane potential of the soma and the available stores. We model the neuron by dividing the morphology into 507 compartments called segments in NEURON (by limiting the maximum length of a compartment to 40 μm). We equip each of these compartments with a single “average-synaptic cleft” model. This simplification allows us to model excess glutamatergic drive that occurs during SD without modeling each of the ~30,000 synapses [39]. Glutamate is released into synapse for the $i^{\text{th}}$ segment at a rate $J_{i}$ when the membrane potential passes a given threshold $v_{crit}$.


$$\begin{array}{c} J_{i}=\left\{ \begin{array}{cc} R_{max}n_{i}^{syn}\frac{N_{i}}{N_{max}}{\left(\frac{v_{\text{soma}}-v_{crit}}{v_{hi}-v_{crit}}\right)}^{2} & \text{if}v_{\text{soma}}>=v_{crit}\\ 0 & \text{otherwise} \end{array}\right. \end{array}$$ (1)

Here $N_{max}$ is the maximum amount (fmol) of stored glutamate and $N_{i}$ is the current store for the $i^{\text{th}}$ section. $R_{max}$ is the maximum release rate, $v_{crit}$ and $v_{hi}$ are the range of membrane potentials where glutamate is released, and $n_{i}^{syn}$ is the number of synapses on the segment. Release of glutamate only depends on the somatic membrane potential.

Uptake occurs from both the cleft and the extracellular space (ECS) and follows Michaelis-Menten kinetics. Glutamate can diffuse between the ECS and the synapses. Glutamate is recycled, replenishing the stored glutamate and ($N_{i}$). N-methyl-D-aspartate receptors (NMDAR) and α -amino-3-hydroxy-5-methyl-4-isoxazolepropionic acid receptors (AMPAR) were also adapted from [20], with NMDAR Ca^2+^ currents based on [65].

### 4.3. Extracellular concentrations and clearance

We simulated diffusion of molecules (K^+^, Na^+^, Cl^-^, Ca^2+^, O_2_ and glutamate) in the ECS bound by the neuron’s convex hull, as approximated with Delaunay tessellation [66]. The hull is required to avoid modeling an unrealistically large empty volume of ECS. The effective free volume was reduced by a factor of 5, since multiple neurons would pass through this region, but we were only modeling a single neuron within this space. Despite this scaling, the model does not capture the observed 20% ECS, 60% intracellular space, and 10% occupied space. To do so would require simulating ~10,000 neurons. Our simulation of the ECS utilizes Neumann (zero flux) boundary conditions, (so K^+^ does not diffuse out of the model). This boundary models the situation where the neuron and glia sit within a larger volume of tissue experiencing identical pathological conditions.

An astrocyte model based on [24,65] with a simplified morphology is included. The same homeostatic mechanisms that feature in the neuron model are included in the astrocyte.

### 4.4. Calcium dynamics

The original model [18] included simple calcium decay in the two radial shells. It used different parameters for the soma, axon, apical dendrites, and basal dendrites, with the $I_{h}$ and K_*A*_ conductances and the passive leak potential dependent on their distance from the soma. Here, the passive leak was replaced with ion-specific leaks independent of distance from the soma.

We expanded this model to include mechanisms supporting calcium-induced-calcium release from [67] (ModelDB: 185858): a calcium buffer, IP_3_ receptors, Ryanodine receptors, and a simplified pathway that increases IP_3_ concentrations in response to metabotropic glutamate receptor (mGluR) activation. We simplified the mGluR mechanisms; rather than modeling the signaling pathway, we only included the essential feature that IP_3_ increases in response to glutamate binding to mGluR.

The Na^+^-Ca^2+^ exchange is an important mechanism in maintaining homeostasis and is implicated in excitotoxicity. Here, we use a ping-pong bi-bi cyclic model and parameters from the mouse cardiac myocyte [68] as the NCX1 from of the Na^+^-Ca^2+^ exchange is expressed both in the heart and brain. The model has a 3:1 stoichiometry and reversal potential of −53 mV (with the initial ion concentrations), closer to the resting membrane potential (RMP) of the neuron −69.5 mV, than the alternatives. The Na^+^-Ca^2+^ exchange model has parameters for saturation with each intracellular and extracellular ion, making this scheme more appropriate for simulating pathology. The density of the Na^+^-Ca^2+^ exchange or its maximum velocity was determined by parameter optimization.

### 4.5. Distance dependent mechanisms

A-type channel (KA) and the HCN $I_{h}$ channel conductances are known to increase with distance from the soma in pyramidal cells [69]. We have incorporated this phenomenon into the model by having $I_{h}$ channel conductance increase linearly with slope 0.03 and KA conductance governed by a sigmoid with length scale 50 μm based on the path distance to the center of the soma.

The $I_{h}$ is permeable to Na^+^ and K^+^ with a relative permeability of 0.36 [70,71]. We used this and the Goldman–Hodgkin–Katz equation to divide the current between the two cations while keeping the same gating dynamics [18].

### 4.6. Parameter fitting

Following the protocols in [18] the model parameters were optimized to reproduce 12 electrophysiological features and to ensure ionic homeostasis in the soma, an apical, and a basal section in response to two stimiuli cases. To keep fits physiologically plausible, any tests with negative conductance values were assigned the worst possible fitness score; no simulations were run in this case. To speed up parameter optimization, the three mechanisms that alter the intracellular chloride concentration (NKCC1, KCC2, and the ion-specific leak) were combined to ensure a non-negative leak conductance for each parameter set.

Optimizations were performed using BluePyOpt [72] with over 600 generations, with a population of 256 parameter sets per generation over 150,000 simulations in total, each taking ~23 minutes for each second of simulated time on a single core of an Intel Xeon E5.

In [18], parameters for the apical dendrites, basal dendrites, and the soma were allowed to vary independently. In this work, to understand the role of morphology and reduce the search space for plausible models, most ion channel conductances were the same between the apical and the basal dendrites. The exceptions were $I_{h}$ and k_*A*_, which depended on the distance from the soma [70], the glutamate mechanism, NMDAR, and AMPAR, which were more densely distributed in the apical dendrites [39].

### 4.7. Human tissue neuropathologic evaluation

Deidentified archival tissue blocks of adult human brain from the Yale Department of Pathology were reviewed by a neuropathologist and sections containing subacute infarct were selected for further workup. Sections were stained using immunohistochemistry with a primary antibody against neurofilament (Clone 2F11, Agilent Inc, Catalog #GA60761–2) and hematoxylin counterstain. Slides were imaged on a Motic EasyScan Infinity 60 imaging system (Motic Inc., Kowloon, Hong Kong) at 40X magnification, with 0.26 μm/pixel resolution. The resulting images were reviewed in QuPath [73] and ROIs containing features of subacute infarct, including loss of parenchymal integrity, reactive astrogliosis, and macrophage infiltration, were selected for further review. Within these regions, intact neurites were identified and evaluated for varicosity.

## Supporting information

S1 FigSlow astrocytic depolarization in response to hypoxia or elevated extracellular K^+^.(A) Elevated extracellular K^+^ led to initial increase in astrocytic ${\text{V}}_{m}$ due to the shift in ${\text{E}}_{K}$. (B) This was followed by a slow, sustained depolarization, which increased slightly when the energy (ATP) was exhausted. (C) Under hypoxic conditions, the astrocyte slowly depolarized, with a rise in ${\text{V}}_{m}$ when ATP was exhausted. A second smaller increase followed, in response to elevated K^+^ when the neuron depolarized. The states for an astrocytic compartment near the neuron’s soma are shown, with similar responses throughout the simplified astrocyte.(TIFF)

S2 FigLower extracellular Ca^2+^ led to reduced intracellular accumulation of Ca^2+^ with a similar distant-dependent pattern.Distribution of ${\text{Ca}}_{cyt}^{2+}$ following 60 s simulation with 0.2 mM extracellular Ca^2+^. (A) Elevated $[K^{+}{]}_{o}$ led to similar pattern of ${\text{Ca}}_{cyt}^{2+}$ accumulation, but with lower concentration (Fig 4A). (B) Under hypoxic conditions ${\text{Ca}}_{cyt}^{2+}$ was most elevated in proximal apical dendrites (0.47 mM to 0.30 mM) with similar concentration in the soma (0.44 mM), and basal dendrites (0.44 mM - 0.38 mM) (Fig 4E). (C) With elevated $[K^{+}{]}_{o}$, ${\text{Ca}}_{cyt}^{2+}$ peaks in the basal dendrites (0.45 mM at 73 μm) and drops in apical dendrites (0.38 mM to 0.08 mM) (Fig 4C). (D) Under hypoxic conditions, ${\text{Ca}}_{cyt}^{2+}$ peaks in apical dendrites near the soma (0.53 mM at 213 μm) and drops with distance from the soma (0.47 mM to 0.30 mM), with similarly high concentration in the soma (0.44 mM) and throughout the basal dendrites (0.44 mM to 0.38 mM) (Fig 4G).(TIFF)

S3 Fig Osmotic pressure. (A, C) With elevated K^+^, the additional extracellular ions dominate the osmotic pressure. (B, D) Under hypoxic conditions, the osmotic pressure drops with distance from the soma. States taken at the end of a 60 s simulation, with an assumed additional 128.7 mM of intracellular anions to give net zero pressure at rest.
(TIFF)

S1 Text Additional analysis of astrocyte dynamics, calcium accumulation, and osmotic pressure. (PDF)

## References

[1] Hall CN, Klein-Flügge MC, Howarth C, Attwell D. Oxidative phosphorylation, not glycolysis, powers presynaptic and postsynaptic mechanisms underlying brain information processing. J Neurosci. 2012;32(26):8940–51. doi: 10.1523/JNEUROSCI.0026-12.2012 22745494 PMC3390246

[2] Attwell D, Laughlin SB. An energy budget for signaling in the grey matter of the brain. J Cereb Blood Flow Metab. 2001;21(10):1133–45. doi: 10.1097/00004647-200110000-00001 11598490

[3] Dreier JP. The role of spreading depression, spreading depolarization and spreading ischemia in neurological disease. Nat Med. 2011;17(4):439–47. doi: 10.1038/nm.2333 21475241

[4] Risher WC, Ard D, Yuan J, Kirov SA. Recurrent spontaneous spreading depolarizations facilitate acute dendritic injury in the ischemic penumbra. J Neurosci. 2010;30(29):9859–68. doi: 10.1523/JNEUROSCI.1917-10.2010 20660268 PMC2918261

[5] Somjen GG. Ions in the brain: normal function, seizures, and stroke. Oxford University Press. 2004.

[6] Somjen GG. Mechanisms of spreading depression and hypoxic spreading depression-like depolarization. Physiol Rev. 2001;81(3):1065–96. doi: 10.1152/physrev.2001.81.3.1065 11427692

[7] Watanabe M, Fukuda A. Development and regulation of chloride homeostasis in the central nervous system. Front Cell Neurosci. 2015;9:371. doi: 10.3389/fncel.2015.00371 26441542 PMC4585146

[8] Drenckhahn C, Winkler MKL, Major S, Scheel M, Kang E-J, Pinczolits A, et al. Correlates of spreading depolarization in human scalp electroencephalography. Brain. 2012;135(Pt 3):853–68. doi: 10.1093/brain/aws010 22366798 PMC3286336

[9] Hartings JA, Shuttleworth CW, Kirov SA, Ayata C, Hinzman JM, Foreman B, et al. The continuum of spreading depolarizations in acute cortical lesion development: Examining Leão’s legacy. J Cereb Blood Flow Metab. 2017;37(5):1571–94. doi: 10.1177/0271678X16654495 27328690 PMC5435288

[10] Dreier JP, Reiffurth C. The stroke-migraine depolarization continuum. Neuron. 2015;86(4):902–22. doi: 10.1016/j.neuron.2015.04.004 25996134

[11] Dreier JP, Fabricius M, Ayata C, Sakowitz OW, Shuttleworth CW, Dohmen C, et al. Recording, analysis, and interpretation of spreading depolarizations in neurointensive care: Review and recommendations of the COSBID research group. J Cereb Blood Flow Metab. 2017;37(5):1595–625. doi: 10.1177/0271678X16654496 27317657 PMC5435289

[12] Gorji A. Spreading depression: a review of the clinical relevance. Brain Res Brain Res Rev. 2001;38(1–2):33–60. doi: 10.1016/s0165-0173(01)00081-9 11750926

[13] Steffensen AB, Sword J, Croom D, Kirov SA, MacAulay N. Chloride Cotransporters as a Molecular Mechanism underlying Spreading Depolarization-Induced Dendritic Beading. J Neurosci. 2015;35(35):12172–87. doi: 10.1523/JNEUROSCI.0400-15.2015 26338328 PMC4556786

[14] Hellas JA, Andrew RD. Neuronal Swelling: A Non-osmotic Consequence of Spreading Depolarization. Neurocrit Care. 2021;35(Suppl 2):112–34. doi: 10.1007/s12028-021-01326-w 34498208 PMC8536653

[15] Hoskison MM, Yanagawa Y, Obata K, Shuttleworth CW. Calcium-dependent NMDA-induced dendritic injury and MAP2 loss in acute hippocampal slices. Neuroscience. 2007;145(1):66–79. doi: 10.1016/j.neuroscience.2006.11.034 17239543 PMC1853289

[16] Hoskison MM, Shuttleworth CW. Microtubule disruption, not calpain-dependent loss of MAP2, contributes to enduring NMDA-induced dendritic dysfunction in acute hippocampal slices. Exp Neurol. 2006;202(2):302–12. doi: 10.1016/j.expneurol.2006.06.010 16904106

[17] Hasbani MJ, Hyrc KL, Faddis BT, Romano C, Goldberg MP. Distinct roles for sodium, chloride, and calcium in excitotoxic dendritic injury and recovery. Exp Neurol. 1998;154(1):241–58. doi: 10.1006/exnr.1998.6929 9875285

[18] Migliore R, Lupascu CA, Bologna LL, Romani A, Courcol J-D, Antonel S, et al. The physiological variability of channel density in hippocampal CA1 pyramidal cells and interneurons explored using a unified data-driven modeling workflow. PLoS Comput Biol. 2018;14(9):e1006423. doi: 10.1371/journal.pcbi.1006423 30222740 PMC6160220

[19] Neymotin SA, McDougal RA, Hines M, Lytton WW. Calcium regulation of HCN supports persistent activity associated with working memory: a multiscale model of prefrontal cortex. BMC Neurosci. 2014;15(S1). doi: 10.1186/1471-2202-15-s1-p108 PMC4724569 26746357

[20] Hübel N, Hosseini-Zare MS, Žiburkus J, Ullah G. The role of glutamate in neuronal ion homeostasis: A case study of spreading depolarization. PLoS Comput Biol. 2017;13(10):e1005804. doi: 10.1371/journal.pcbi.1005804 29023523 PMC5655358

[21] Wei Y, Ullah G, Schiff SJ. Unification of neuronal spikes, seizures, and spreading depression. J Neurosci. 2014;34(35):11733–43. doi: 10.1523/JNEUROSCI.0516-14.2014 25164668 PMC4145176

[22] Le Masson G, Przedborski S, Abbott LF. A computational model of motor neuron degeneration. Neuron. 2014;83(4):975–88. doi: 10.1016/j.neuron.2014.07.001 25088365 PMC4167823

[23] Somjen GG, Kager H, Wadman WJ. Calcium sensitive non-selective cation current promotes seizure-like discharges and spreading depression in a model neuron. J Comput Neurosci. 2009;26(1):139–47. doi: 10.1007/s10827-008-0103-9 18563545

[24] Kager H, Wadman WJ, Somjen GG. Conditions for the triggering of spreading depression studied with computer simulations. J Neurophysiol. 2002;88(5):2700–12. doi: 10.1152/jn.00237.2002 12424305

[25] Kager H, Wadman WJ, Somjen GG. Simulated seizures and spreading depression in a neuron model incorporating interstitial space and ion concentrations. J Neurophysiol. 2000;84(1):495–512. doi: 10.1152/jn.2000.84.1.495 10899222

[26] Hamill OP, Marty A, Neher E, Sakmann B, Sigworth FJ. Improved patch-clamp techniques for high-resolution current recording from cells and cell-free membrane patches. Pflugers Arch. 1981;391(2):85–100. doi: 10.1007/BF00656997 6270629

[27] Perkins KL. Cell-attached voltage-clamp and current-clamp recording and stimulation techniques in brain slices. J Neurosci Methods. 2006;154(1–2):1–18. doi: 10.1016/j.jneumeth.2006.02.010 16554092 PMC2373773

[28] Harris JP, Mietus CJ, Browne KD, Wofford KL, Keating CE, Brown DP, et al. Neuronal somatic plasmalemmal permeability and dendritic beading caused by head rotational traumatic brain injury in pigs-An exploratory study. Front Cell Neurosci. 2023;17:1055455. doi: 10.3389/fncel.2023.1055455 37519631 PMC10381956

[29] Swann JW, Al-Noori S, Jiang M, Lee CL. Spine loss and other dendritic abnormalities in epilepsy. Hippocampus. 2000;10(5):617–25. doi: 10.1002/109e8-1063(2000)10:5<617::AID-HIPO13>3.0.CO;2-R 11075833

[30] Hori N, Carpenter DO. Functional and morphological changes induced by transient in vivo ischemia. Exp Neurol. 1994;129(2):279–89. doi: 10.1006/exnr.1994.1170 7957741

[31] Murphy TH, Li P, Betts K, Liu R. Two-photon imaging of stroke onset in vivo reveals that NMDA-receptor independent ischemic depolarization is the major cause of rapid reversible damage to dendrites and spines. J Neurosci. 2008;28(7):1756–72. doi: 10.1523/JNEUROSCI.5128-07.2008 18272696 PMC6671530

[32] Rizzuto R, Pinton P, Ferrari D, Chami M, Szabadkai G, Magalhães PJ, et al. Calcium and apoptosis: facts and hypotheses. Oncogene. 2003;22(53):8619–27. doi: 10.1038/sj.onc.1207105 14634623

[33] Kager H, Wadman WJ, Somjen GG. Seizure-like afterdischarges simulated in a model neuron. J Comput Neurosci. 2007;22(2):105–28. doi: 10.1007/s10827-006-0001-y 17053996

[34] Martínez-Sánchez M, Striggow F, Schröder UH, Kahlert S, Reymann KG, Reiser G. Na(+) and Ca(2+) homeostasis pathways, cell death and protection after oxygen-glucose-deprivation in organotypic hippocampal slice cultures. Neuroscience. 2004;128(4):729–40. doi: 10.1016/j.neuroscience.2004.06.074 15464281

[35] Calabresi P, Marfia GA, Centonze D, Pisani A, Bernardi G. Sodium influx plays a major role in the membrane depolarization induced by oxygen and glucose deprivation in rat striatal spiny neurons. Stroke. 1999;30(1):171–9. doi: 10.1161/01.str.30.1.171 9880406

[36] Yellen G. Fueling thought: Management of glycolysis and oxidative phosphorylation in neuronal metabolism. J Cell Biol. 2018;217(7):2235–46. doi: 10.1083/jcb.201803152 29752396 PMC6028533

[37] Boumezbeur F, Petersen KF, Cline GW, Mason GF, Behar KL, Shulman GI, et al. The contribution of blood lactate to brain energy metabolism in humans measured by dynamic 13C nuclear magnetic resonance spectroscopy. J Neurosci. 2010;30(42):13983–91. doi: 10.1523/JNEUROSCI.2040-10.2010 20962220 PMC2996729

[38] Wu K, Aoki C, Elste A, Rogalski-Wilk AA, Siekevitz P. The synthesis of ATP by glycolytic enzymes in the postsynaptic density and the effect of endogenously generated nitric oxide. Proc Natl Acad Sci U S A. 1997;94(24):13273–8. doi: 10.1073/pnas.94.24.13273 9371836 PMC24299

[39] Megías M, Emri Z, Freund TF, Gulyás AI. Total number and distribution of inhibitory and excitatory synapses on hippocampal CA1 pyramidal cells. Neuroscience. 2001;102(3):527–40. doi: 10.1016/s0306-4522(00)00496-6 11226691

[40] Bannister NJ, Larkman AU. Dendritic morphology of CA1 pyramidal neurones from the rat hippocampus: I. Branching patterns. J Comp Neurol. 1995;360(1):150–60. doi: 10.1002/cne.903600111 7499560

[41] Ropireddy D, Bachus SE, Ascoli GA. Non-homogeneous stereological properties of the rat hippocampus histological sections. Neuroscience. 2012;205:91–111. doi: 10.1016/j.neuroscience.2011.12.055 22245503 PMC3299877

[42] Grassi D, Idziak A, Lee A, Calaresu I, Sibarita J-B, Cognet L, et al. Nanoscale and functional heterogeneity of the hippocampal extracellular space. Cell Rep. 2023;42(5):112478. doi: 10.1016/j.celrep.2023.112478 37149864 PMC10242443

[43] Shaw K, Bell L, Boyd K, Grijseels DM, Clarke D, Bonnar O, et al. Neurovascular coupling and oxygenation are decreased in hippocampus compared to neocortex because of microvascular differences. Nat Commun. 2021;12(1):3190. doi: 10.1038/s41467-021-23508-y 34045465 PMC8160329

[44] Weilinger NL, Wicki-Stordeur LE, Groten CJ, LeDue JM, Kahle KT, MacVicar BA. KCC2 drives chloride microdomain formation in dendritic blebbing. Cell Rep. 2022;41(4):111556. doi: 10.1016/j.celrep.2022.111556 36288701

[45] Mestre H, Du T, Sweeney AM, Liu G, Samson AJ, Peng W, et al. Cerebrospinal fluid influx drives acute ischemic tissue swelling. Science. 2020;367(6483):eaax7171. doi: 10.1126/science.aax7171 32001524 PMC7375109

[46] Herreras O, Largo C, Ibarz JM, Somjen GG, Martín del Río R. Role of neuronal synchronizing mechanisms in the propagation of spreading depression in the in vivo hippocampus. J Neurosci. 1994;14(11 Pt 2):7087–98. doi: 10.1523/JNEUROSCI.14-11-07087.1994 7965100 PMC6577274

[47] Dietz RM, Weiss JH, Shuttleworth CW. Zn2+ influx is critical for some forms of spreading depression in brain slices. J Neurosci. 2008;28(32):8014–24. doi: 10.1523/JNEUROSCI.0765-08.2008 18685026 PMC2577031

[48] Kelley C, Newton AJH, Hrabetova S, McDougal RA, Lytton WW. Multiscale Computer Modeling of Spreading Depolarization in Brain Slices. eNeuro. 2022;9(4). doi: 10.1523/ENEURO.0082-22.2022 PMC9410770 35927026

[49] Silver IA, Erecińska M. Intracellular and extracellular changes of [Ca2+] in hypoxia and ischemia in rat brain in vivo. J Gen Physiol. 1990;95(5):837–66. doi: 10.1085/jgp.95.5.837 2163431 PMC2216343

[50] Kristián T, Siesjö BK. Calcium in ischemic cell death. Stroke; a journal of cerebral circulation. 1998;29(3):705–18. doi: 10.1161/01.str.29.3.705 9506616

[51] Lee A, Kondapalli C, Virga DM, Lewis TL Jr, Koo SY, Ashok A, et al. Aβ42 oligomers trigger synaptic loss through CAMKK2-AMPK-dependent effectors coordinating mitochondrial fission and mitophagy. Nat Commun. 2022;13(1):4444. doi: 10.1038/s41467-022-32130-5 35915085 PMC9343354

[52] Pérez-Pinzón MA, Tao L, Nicholson C. Extracellular potassium, volume fraction, and tortuosity in rat hippocampal CA1, CA3, and cortical slices during ischemia. J Neurophysiol. 1995;74(2):565–73. doi: 10.1152/jn.1995.74.2.565 7472364

[53] Larsen GA, Skjellegrind HK, Moe MC, Vinje ML, Berg-Johnsen J. Endoplasmic reticulum dysfunction and Ca2+ deregulation in isolated CA1 neurons during oxygen and glucose deprivation. Neurochem Res. 2005;30(5):651–9. doi: 10.1007/s11064-005-2753-6 16176069

[54] Bartsch T, Döhring J, Reuter S, Finke C, Rohr A, Brauer H, et al. Selective neuronal vulnerability of human hippocampal CA1 neurons: lesion evolution, temporal course, and pattern of hippocampal damage in diffusion-weighted MR imaging. J Cereb Blood Flow Metab. 2015;35(11):1836–45. doi: 10.1038/jcbfm.2015.137 26082014 PMC4635239

[55] Hines M, Carnevale T, McDougal RA. NEURON Simulation Environment. In: Jaeger R, Dieterand J. Encyclopedia of Computational Neuroscience. New York, NY: Springer New York. 2019. p. 1–7. doi: 10.1007/978-1-4614-7320-6_795-2

[56] Carnevale NT, Hines ML. The NEURON book. Cambridge University Press. 2006.

[57] McDougal RA, Hines ML, Lytton WW. Reaction-diffusion in the NEURON simulator. Frontiers in Neuroinformatics. 2013;7. 10.3389/fninf.2013.00028 PMC3828620 24298253

[58] Newton AJH, McDougal RA, Hines ML, Lytton WW. Using NEURON for Reaction-Diffusion Modeling of Extracellular Dynamics. Front Neuroinform. 2018;12:41. doi: 10.3389/fninf.2018.00041 30042670 PMC6049079

[59] Veech RL, Lawson JW, Cornell NW, Krebs HA. Cytosolic phosphorylation potential. J Biol Chem. 1979;254(14):6538–47. doi: 10.1016/s0021-9258(18)50401-4 36399

[60] Rich PR. The molecular machinery of Keilin’s respiratory chain. Biochem Soc Trans. 2003;31(Pt 6):1095–105. doi: 10.1042/bst0311095 14641005

[61] Hubley MJ, Locke BR, Moerland TS. The effects of temperature, pH, and magnesium on the diffusion coefficient of ATP in solutions of physiological ionic strength. Biochim Biophys Acta. 1996;1291(2):115–21. doi: 10.1016/0304-4165(96)00053-0 8898871

[62] Gerkau NJ, Lerchundi R, Nelson JSE, Lantermann M, Meyer J, Hirrlinger J, et al. Relation between activity-induced intracellular sodium transients and ATP dynamics in mouse hippocampal neurons. J Physiol. 2019;597(23):5687–705. doi: 10.1113/JP278658 31549401

[63] Noske R, Cornelius F, Clarke RJ. Investigation of the enzymatic activity of the Na+,K+-ATPase via isothermal titration microcalorimetry. Biochim Biophys Acta. 2010;1797(8):1540–5. doi: 10.1016/j.bbabio.2010.03.021 20362545

[64] Mahmmoud YA. Capsaicin stimulates uncoupled ATP hydrolysis by the sarcoplasmic reticulum calcium pump. J Biol Chem. 2008;283(31):21418–26. doi: 10.1074/jbc.M803654200 18539598

[65] Somjen GG, Kager H, Wadman WJ. Computer simulations of neuron-glia interactions mediated by ion flux. J Comput Neurosci. 2008;25(2):349–65. doi: 10.1007/s10827-008-0083-9 18297383

[66] Virtanen P, Gommers R, Oliphant TE, Haberland M, Reddy T, Cournapeau D, et al. SciPy 1.0: fundamental algorithms for scientific computing in Python. Nat Methods. 2020;17(3):261–72. doi: 10.1038/s41592-019-0686-2 32015543 PMC7056644

[67] Neymotin SA, McDougal RA, Bulanova AS, Zeki M, Lakatos P, Terman D, et al. Calcium regulation of HCN channels supports persistent activity in a multiscale model of neocortex. Neuroscience. 2016;316:344–66. doi: 10.1016/j.neuroscience.2015.12.043 26746357 PMC4724569

[68] Weber CR, Ginsburg KS, Philipson KD, Shannon TR, Bers DM. Allosteric regulation of Na/Ca exchange current by cytosolic Ca in intact cardiac myocytes. J Gen Physiol. 2001;117(2):119–31. doi: 10.1085/jgp.117.2.119 11158165 PMC2217247

[69] Migliore M, Shepherd GM. Emerging rules for the distributions of active dendritic conductances. Nat Rev Neurosci. 2002;3(5):362–70. doi: 10.1038/nrn810 11988775

[70] Magee JC. Dendritic hyperpolarization-activated currents modify the integrative properties of hippocampal CA1 pyramidal neurons. J Neurosci. 1998;18(19):7613–24. doi: 10.1523/JNEUROSCI.18-19-07613.1998 9742133 PMC6793032

[71] Aponte Y, Lien C-C, Reisinger E, Jonas P. Hyperpolarization-activated cation channels in fast-spiking interneurons of rat hippocampus. J Physiol. 2006;574(Pt 1):229–43. doi: 10.1113/jphysiol.2005.104042 16690716 PMC1817792

[72] Van Geit W, Gevaert M, Chindemi G, Rössert C, Courcol J-D, Muller EB, et al. BluePyOpt: Leveraging Open Source Software and Cloud Infrastructure to Optimise Model Parameters in Neuroscience. Front Neuroinform. 2016;10:17. doi: 10.3389/fninf.2016.00017 27375471 PMC4896051

[73] Bankhead P, Loughrey MB, Fernández JA, Dombrowski Y, McArt DG, Dunne PD, et al. QuPath: Open source software for digital pathology image analysis. Sci Rep. 2017;7(1):16878. doi: 10.1038/s41598-017-17204-5 29203879 PMC5715110

