# Supplementary material for: Computer models predict differential dendritic vulnerability with ischemia and spreading depression
Source: PLoS Comput Biol. 2026 Sep 8;22(9):e1014701. doi: 10.1371/journal.pcbi.1014701 (PMC13568529; doi:10.1371/journal.pcbi.1014701)
Supplement: Supplementary file 1 [file pcbi.1014701.s004.pdf]

S1 Text

Astrocyte dynamics

Astrocytes clear extracellular K^+ and glutamate when sufficient energy is available. With elevated extracellular K^+ (Fig 3A - 3C), our model showed an initial increase in astrocytic V_m (S1A Fig) followed by a slow sustained depolarization (S1B Fig). Under hypoxic conditions (Fig 3D - 3F), there were relatively large changes in astrocytic V_m when ATP was exhausted and when the neuron depolarized (S1C Fig). In both cases, the changes in astrocytic V_m were smaller than those in the neuron.

Reduced extracellular calcium leads to reduced intracellular levels

Hypoxic and elevated extracellular K^+ simulations, with reduced extracellular Ca^{2+}, led to a similar loss of ion homeostasis but with a reduced increase in Ca^{2+}_{cyt}. The loss of energy for the PMCA or SERCA and more significantly the loss of the Na^+ gradient to drive Na^+-Ca^{2+} exchange, led to Ca^{2+}_{cyt} almost equilibrating with extracellular Ca^{2+}.

Osmotic pressure

Excess extracellular pressure (negative osmotic pressure) affects most of the neuron (S3 Fig). Ignoring ion-ion interactions, we used the Van 't Hoff formula $\Pi_i = \mathrm{RT} \sum_{x \in \mathrm{ions}} [X]_i$ and $\Pi_o = \mathrm{RT} \sum_{x \in \mathrm{ions}} [X]_o$ for the intracellular and extracellular osmotic pressure, where T is the temperature and R is the gas constant. The pressure difference is given by $\Delta\Pi = \Pi_o - \Pi_i$. We assumed an additional concentration of 128.7 mM of intracellular impermeable anions, so the initial osmotic pressure difference is zero. With elevated extracellular K^+, the pressure is dominated by the additional K^+ (S3A Fig and S3C Fig). Under hypoxic conditions, the magnitude of the osmotic pressure difference drops with distance from the soma (S3B Fig and S3D Fig). This is a result of higher extracellular ion concentrations around the soma and basal dendrites. However, water entry into the cell is not a purely osmotic event and it depends on Cl^- [13]. Volume dynamics are not included in the model. Shrinkage of the ECS would increase extracellular concentrations and swelling of the neuron would reduce intracellular concentrations, both acting to reduce the osmotic difference.
